# Opportunistic pathogens and large microbial diversity detected in source-to-distribution drinking water of three remote communities in Northern Australia

**DOI:** 10.1371/journal.pntd.0007672

**Published:** 2019-09-05

**Authors:** Mirjam Kaestli, Michelle O’Donnell, Alea Rose, Jessica R. Webb, Mark Mayo, Bart J. Currie, Karen Gibb

**Affiliations:** 1 Research Institute for the Environment and Livelihoods, Charles Darwin University, Darwin, Northern Territory, Australia; 2 Global and Tropical Health, Menzies School of Health Research, Darwin, Northern Territory, Australia; 3 Power and Water Corporation, Darwin, Northern Territory, Australia; University of Texas Medical Branch, UNITED STATES

## Abstract

In the wet-dry tropics of Northern Australia, drinking water in remote communities is mostly sourced from bores accessing groundwater. Many aquifers contain naturally high levels of iron and some are shallow with surface water intrusion in the wet season. Therefore, environmental bacteria such as iron-cycling bacteria promoting biofilm formation in pipes or opportunistic pathogens can occur in these waters. An opportunistic pathogen endemic to northern Australia and Southeast Asia and emerging worldwide is *Burkholderia pseudomallei*. It causes the frequently fatal disease melioidosis in humans and animals. As we know very little about the microbial composition of drinking water in remote communities, this study aimed to provide a first snapshot of the microbiota and occurrence of opportunistic pathogens in bulk water and biofilms from the source and through the distribution system of three remote water supplies with varying iron levels. Using 16s-rRNA gene sequencing, we found that the geochemistry of the groundwater had a substantial impact on the untreated microbiota. Different iron-cycling bacteria reflected differences in redox status and nutrients. We cultured and sequenced *B*. *pseudomallei* from bores with elevated iron and from a multi-species biofilm which also contained iron-oxidizing *Gallionella*, nitrifying *Nitrospira* and amoebae. *Gallionella* are increasingly used in iron-removal filters in water supplies and more research is needed to examine these interactions. Similar to other opportunistic pathogens, *B*. *pseudomallei* occurred in water with low organic carbon levels and with low heterotrophic microbial growth. No *B*. *pseudomallei* were detected in treated water; however, abundant DNA of another opportunistic pathogen group, non-tuberculous mycobacteria was recovered from treated parts of one supply. Results from this study will inform future studies to ultimately improve management guidelines for water supplies in the wet-dry tropics.

## Author summary

Water providers in the wet-dry tropics of Northern Australia face additional challenges to keep drinking water microbiologically safe. The source water is often rich in iron-cycling bacteria leading to excessive biofilm formation in pipes and it can also contain the emerging opportunistic pathogen *Burkholderia pseudomallei* causing the severe disease melioidosis in humans and animals. We know very little about the ecology of microbes in remote community water supplies, so to start to fill this gap we assessed the microbial composition from the source to the distribution of three remote water supplies. We not only found that the geochemistry of the source water had a substantial impact on the composition of the iron-cycling bacteria but *B*. *pseudomallei* was cultured from source water with low organic carbon but elevated iron levels and from a multi-species biofilm linked to iron bacteria. No *B*. *pseudomallei* were detected in treated water; however, abundant DNA of another opportunistic pathogen group, non-tuberculous mycobacteria, was recovered from treated parts of one water supply. This work lays the foundation for future studies to ultimately improve management guidelines for water supplies in the wet-dry tropics.

## Introduction

Water providers in the wet-dry tropics of Northern Australia face significant challenges to keep drinking water safe and free of opportunistic pathogens. One such opportunistic pathogen is *Burkholderia pseudomallei*, an environmental saprophytic bacterium and causative agent of the severe disease melioidosis affecting humans and animals [1, 2]. People most at risk are those suffering from diabetes, chronic lung or renal disease or hazardous alcohol use [3]. Until recently, melioidosis was thought to mainly affect people in Northern Australia and Southeast Asia where *B*. *pseudomallei* is endemic. However, a recent modelling study predicted 165,000 annual melioidosis cases worldwide of whom 89,000 were estimated to die [1]. *B*. *pseudomallei* is a natural component of the soil and surface water microbiota in rural Darwin, Northern Territory in northern Australia and 30% of tested unchlorinated residential water wells (bores) were positive for the bacteria [4, 5]. *B*. *pseudomallei* has been isolated from aerator sprays and tank sludge from water treatment plants ([6]; own observation) and melioidosis cases and deaths due to contaminated drinking water have been documented in Northern Australia and Thailand [6–10]. These supplies were either not chlorinated or the disinfection process was interrupted. *B*. *pseudomallei* is successfully contained by free chlorine levels of 0.5 to 1 mg/L, although in laboratory experiments, some strains were more chlorine tolerant [11].

Groundwater in many areas of Northern Australia contains naturally high levels of iron and it is unclear to what degree this promotes *B*. *pseudomallei* survival. *B*. *pseudomallei* has a redundant system of siderophores allowing it to acquire non-bioavailable ferric iron [12, 13]; a positive association between *B*. *pseudomallei* and total iron levels was found in bore water [14] while the association was negative in soil with high iron levels [15, 16] suggesting a unimodal rather than linear relationship across the range of iron levels encountered in the environment.

Water with high iron levels attracts naturally occurring iron bacteria which metabolize the iron and contribute to pipe corrosion and reduced bore yield. While some bacteria such as *Geothrix fermentens* or *Shewanella sp*. reduce iron in anoxic groundwater using organic carbon as electron donor, in niches with low oxygen iron oxidizers such as *Gallionella ferruginea* thrive and facilitate the production of abundant ferric oxide precipitates which block pipes, and contribute to biofilm formation reducing disinfection efficiency [17–19]. Most biofilms consist of a complex mix of bacterial taxa and can also be associated with fungi, viruses or protozoa [18]. Although biofilms are a known reservoir for opportunistic pathogens such as nontuberculous Mycobacteria, *Legionella pneumophila* or *Pseudomonas aeruginosa* [20], we still do not know to what degree *B*. *pseudomallei* colonizes multi-species biofilms in water pipes.

Water supplies of remote communities mainly depend on chlorination as disinfection treatment and are vulnerable to exposure to opportunistic pathogens in the event of a chlorination breakdown or if pathogens are chlorine-resistant. Indigenous people in remote communities often have higher rates of chronic diseases such as diabetes and thus, are more at risk of infection if exposed to opportunistic pathogens [21]. A multiple barrier approach to improve water quality is needed [22]. However, without full knowledge of what microbes occur in the source and distribution water, it can be difficult to design and apply barriers suitable for northern Australia.

This scoping study aimed to provide a first snapshot of the microbiota in bulk water and biofilms from the source and through the distribution system in three water supplies of remote communities; one supply with naturally high iron levels, one with medium and one with low levels. There were three study objectives: A) the detection and culture of opportunistic pathogens with a focus on endemic *B*. *pseudomallei*; B) the detection of taxa with known iron bacteria using 16s rRNA gene amplicon sequencing for microbial profiling; and C) the characterisation of the bacterial and archaeal microbiota and its association with nutrients and site characteristics. We hypothesized that water treatment would have the largest impact on the microbiota followed by the origin of the source water. We also hypothesized that water supplies fed from unconfined shallow aquifers would contain more bacteria also occurring in soil including *B*. *pseudomallei*. Results from this work will inform and guide future studies to ultimately improve management guidelines suitable for Northern Australia to minimize microbial risk in the drinking water distribution network.

## Methods

### Sampling sites and time of sampling

Water and biofilms from the drinking water distribution system (DWDS) were sampled from three remote Indigenous communities in the Top End of the Northern Territory (NT), Australia. The Top End has a tropical savannah climate, with a distinct dry and wet season and average annual rainfall of 1,727 mm between Oct and March (www.bom.gov.au). The “HighFe” or HF community had a water supply with high iron (Fe) levels in the source water with median 0.80 mg/L total iron levels. This was above the aesthetic guideline value of 0.30 mg/L of the Australian drinking water guidelines [22]. The “MidFe” or MF community had a water supply with medium iron (Fe) levels with median 0.25 mg/L total iron and the “LowFe” or LF community had low iron levels with a median 0.05 mg/L total iron. All three communities had reported melioidosis cases in the past (1994–2017: HF 3 cases (incidence rate IR 4.1 cases/1,000 population), MF 11 cases (IR 9.9) and LF 4 cases (IR 2.6)). It is not known where these patients acquired the melioidosis bacteria.

Samples were collected in the late wet season i.e. in March 2017 for two of the three communities (HF and LF) while the third community (MF) was sampled in May 2017 as soon as waters receded sufficiently to allow access to the water bore fields. For each community, samples were collected from five points along the DWDS of which three were unchlorinated (bores and tanks) and two from the chlorinated reticulation system.

#### Community HF

The community HF with high iron levels in its water supply had a population of approximately 800 people in 2017 (NT Government) and sourced its drinking water from 11 groundwater bores accessing shallow aquifers in coastal sandy soil. Water in the aquifer is pumped from the bores to ground level storage tanks. To promote the removal of iron, a curtain was installed in one of the ground level water storage tanks to encourage settling of iron prior to disinfection. Sodium hypochlorite was then dosed directly into the water main and the treated water was pumped into another ground level tank for storage before being distributed to the community. The five sample collection sites consisted of an unchlorinated bore (depth 14 metres to an unconfined aquifer in quaternary coastal and Paleochannel deposit, built in 2003, and yields 2 L/sec), water storage tank, rising main pipe and two water meters from the chlorinated reticulation system in the community. The bore field is prone to seasonal flooding during the monsoonal period.

#### Community MF

The community MF with mid-iron levels had a population of approximately 1,400 people in 2017 with 15 operational bores mainly accessing a shallow semi-confined aquifer in quaternary alluvial deposits consisting of sand and gravels. During the wet season the bore field is often inundated, and the groundwater intersection is close to the surface. Water disinfection is by chlorine gas and ultraviolet. The five sites consisted of three bores and two chlorinated water meters from within the community. These bores were built between 1972 and 1984 and the major casing material was steel as opposed to stainless steel for the other bores tested in this study. Their yield ranged between 1.0–1.4 L/sec. Two were shallow bores with a screening depth of less than 10 metres while the third bore was fed by a semi-confined aquifer in Kombolgie sandstone at 17.5–23.5 screening depth.

#### Community LF

The community LF with low iron levels had a population of approximately 1,800 people in 2017 and sourced its water supply from 9 groundwater bores. The bores access an unconfined aquifer in Van Diemen sandstone consisting of fine-grained sandstone with sandy clay horizons. No *E*. *coli* were cultured from the groundwater in the past. Water is treated with chlorine gas and it also contains a fluoridation system. The five sites consisted of three bores and two chlorinated water meters within the community. The three bores were screened at a depth of 38 to 54 metres, built in 2006 with a yield of 1.7–4.2 L/sec.

### Sample collection

Samples were collected from five sites from each of three water supplies. One litre of water was collected in duplicate for subsequent DNA extraction. An additional 500 mL were collected for *B*. *pseudomallei* culture (Menzies School of Health Research), 200 mL in duplicate in 200 mL sodium thiosulphate dosed bottles for subsequent faecal indicator, heterotroph and amoebae culture, 100 mL into acid-washed 125 mL bottles for elemental analysis and 100 mL of *in situ* filtered water (using 0.45 micron filters) into acid-washed 125 mL bottles for nutrient analysis. All bores that were sampled were in operation for >6 hours and bores were purged for five minutes prior to water collection. The surface of biofilms in the bore head, pipes, tanks, and water meter walls was collected in duplicates using sterile swabs (Interpath, Australia). All samples were kept on ice on the sampling day except water and biofilms for subsequent amoebae and *B*. *pseudomallei* culture which were kept at room temperature and protected from sunlight. A total of 60 water and biofilm samples were collected in duplicates from 15 water collection points of three water supplies.

A YSI meter (www.ysi.com) was used to measure various physicochemical factors in water namely pH, salinity, temperature, turbidity and dissolved oxygen (DO) content. A colorimeter was used to measure free chlorine levels of the chlorinated water. A redox meter calibrated with Zobell’s solution (YSI) measured the oxidation redox potential (ORP)–redox measurements were conducted for all samples on the same day of collection upon return to the laboratory.

### Culture of opportunistic pathogens and faecal indicators

*E*. *coli*, coliforms, *P*. *aeruginosa*, heterotrophs and free-living amoebae were cultured at the NATA accredited NT Government Dept. of Primary Industry and Resources laboratory and the Australian Water Quality Centre (AWQC) after overnight shipment of samples on ice (room temperature for amoebae). Culture of *E*. *coli* and coliforms was based on the Most Probable Number (MPN) method and Colilert-18 Defined Substrate Technology (DST) (AS/NZS 4276.21–2005) while culture of *P*. *aeruginosa* was by membrane filtration. Heterotrophic Colony Count was by pour plate method with incubation for 44 h at 36 C (AS 4276.3.1–2007). Culture for *B*. *pseudomallei* and near-neighbour *Burkholderia* was conducted at Menzies School of Health Research. Culture from 500 mL of water was based on membrane filtration (0.22 micron filters) followed by culture in Ashdown broth and agar as previously described [4]. Similarly, biofilm swabs were incubated in Ashdown broth followed by plating on Ashdown agar.

### Whole genome sequencing of *B*. *pseudomallei* isolates

DNA extraction of six *B*. *pseudomallei* isolates was as previously described [23] and the genomes were sequenced on a Illumina HiSeq2500 platform (Illumina, Inc., San Diego, CA) at the Australian Genome Research Facility (AGRF).

### Analysis of *B*. *pseudomallei* whole genome sequences (WGS)

Orthologous core single nucleotide polymorphism (SNP) variants were identified among 89 *B*. *pseudomallei* genomes from the Northern Territory using the default settings of SPANDx v3.2 [24] and the closed Australian *B*. *pseudomallei* genome MSHR1153 [25] as reference (N50 4,032,226 bp; 2 contigs; size 7,312,903 bp). A maximum parsimony phylogenetic tree was generated in PAUP* 4.0.b5 [26] based on 174,905 SNPs and rooted using MSHR668. Multi-locus sequence types (MLST) were assigned *in silico* using the BIGSdb tool which is accessible on the *B*. *pseudomallei* MLST website (http://pubmlst.org/bpseudomallei/). The following geographical and virulence genetic markers were extracted *in silico* using the Basic Local Alignment Search Tool (BLAST) [27] following previously published methods [28]: LPS A (*wbil* to *apaH* in K96243 [GenBank ref: NC_006350]), LPS B (*BUC_3392* to *apaH* in *B*. *pseudomallei* 579 [GenBank ref: NZ_ACCE01000003]), LPS B2 (*BURP840_LPSb01* to *BURP840_LPSb21* in *B*. *pseudomallei* MSHR840 [GenBank ref: GU574442]), BTFC (*lafU* in *B*. *pseudomallei* MSHR668 [GenBank ref: NC_006350]), YLF (*BPSS0124* in *B*. *pseudomallei* K96243 [GenBank ref: CP009545.1]), *bimA*_*Bm*_ (*BURPS668_A2118* in *B*. *pseudomallei* MSHR668 [GenBank ref: NZ_CP009545]), *bimA*_*Bp*_ (*BPSS1492* in *B*. *pseudomallei* K96243 [GenBank ref: NC_006350]) and *fhaB3* (*BPSS2053* in *B*. *pseudomallei* K96243 [GenBank ref: NC_006350]).

### Element and nutrient analysis

Elements (total Fe Mn Mo Mg K Ca S Ni Cu Zn) were measured at the Environmental Chemistry & Microbiology Unit (ECMU) (CDU, Darwin, Australia) by ICP-MS (AGILENT 7700ce, www.agilent.com)[29]. Dissolved nutrient analysis (TDN, NOx, TDP and DOC) of the filtered water was conducted at the laboratory of Queensland Health (www.health.qld.gov.au).

### Water and biofilm DNA extraction

Within 24h of collection, water samples (1 L) were filtered (0.45 micron filters, Sartorius) and frozen until processed. DNA was extracted from filters and swabs using the FastDNA soil kit (MPBio, Australia) following the manufacturers’ instructions. Bacterial load was measured using a SYBR-based qPCR assay targeting the 16s rRNA gene with PCR primers 331-f and 797-r [30] and using the QuantiTect SYBR Green qPCR mix (Qiagen, Australia) resulting in a qPCR efficiency of 90%. The delta Ct method was used for relative quantification and a positive control was included in each run for inter-run comparisons. Five DNA extraction negative controls on filters (#3) and swabs (#2) with no water or biofilm added were also processed. The DNA was sent to the Australian Centre for Ecogenomics (ACE, https://ecogenomic.org/) for 16s rRNA gene amplicon sequencing.

### Sixteen s rRNA gene sequencing

Sixteen-s rRNA gene amplification and Illumina MiSeq sequencing was conducted at ACE using the Earth Microbiome Project 16s rRNA V4 515FB-806RB universal primers (FWD:GTGYCAGCMGCCGCGGTAA; REV:GGACTACNVGGGTWTCTAAT) targeting bacteria and archaea (accessed June 2017: http://press.igsb.anl.gov/earthmicrobiome/protocols-and-standards/16s/). These primers were extensively validated to minimize bias towards or against taxonomic groups; however, remaining preferential amplification of certain taxa cannot be excluded [31–33]. Sequences were processed to sequence variants (SVs) by ACE with the following pipeline. The software Trimmomatic was used for sequence quality trimming removing poor quality sequences with a sliding window of 4 bases and an average base quality above 15. All reads were hard trimmed to 250 bases, and any with less excluded. Reads were processed to SVs using the QIIME-2 workflow with default parameters and the DADA-2 algorithm [34, 35]. Taxonomic assignment of SVs was through BLAST+ using the reference database SILVA (www.arb-silva.de).

### Processing 16s rRNA gene sequence variant data

15,590 SVs were further processed using the R library Phyloseq. Due to the low biomass of many samples, special care was taken to exclude potential contaminant SVs such as from lab reagents [36, 37]. Seventeen SVs were excluded which consistently occurred in all five negative controls. A further five SVs were excluded which occurred in minimum two negative controls and showed a significant negative Spearman’s rank correlation with the bacterial DNA abundance based on 16s qPCR results (P<0.05). The R package “decontam” was used to exclude a further 93 SVs based on their occurrence in negative controls (prevalence method). Thus, a total of 104 SVs were excluded due to contamination concerns. A further 9,787 SVs (63%) were excluded as these only occurred in one sample (with duplicate water and biofilm samples collected per site). As a last step, 38 SVs were excluded as these were not assigned to either Bacteria or Archaea.

Nineteen of 60 samples (32%) were excluded due to low sequence counts (< 5,000 sequences) (7 chlorinated water, 6 unchlorinated water, 4 chlorinated biofilms, 2 unchlorinated biofilms). Negative control samples had sequence counts ranging between 334 and 1,840 sequences. A hierarchical cluster analysis was performed in Primer-E v7 (Plymouth, UK) to test whether samples clustered with negative controls. This was only the case for samples with low sequence counts which were excluded from any downstream analyses. The final dataset contained 5,411 different SVs and 41 samples. All remaining samples were rarefied to the lowest common sequence number per sample (5,259 sequences). Rarefaction curves indicated that with this cut-off, the richness of all chlorinated samples plateaued i.e. was reached; however, four of 16 unchlorinated biofilm samples (25%) and two of 12 unchlorinated water samples (17%) only reached approximately 70–80% of their SV richness (S1 Fig). To examine Mycobacteria counts and richness across primarily chlorinated samples, a lower rarefaction threshold of 2,000 sequences was adopted which allowed the inclusion of more chlorinated samples (22 of 24 chlorinated samples) while their full SV richness was reached at this threshold, which was still higher than the sequencing depth of all negative controls (S1 Fig).

### Biostatistical data analysis

The weighted UniFrac and Bray Curtis distance matrices were trialled on the rarefied and square-root transformed SV data. Non-metric multidimensional scaling ordinations (nMDS) on the weighted UniFrac matrices showed a high stress value (>0.2) and therefore, the Bray Curtis dissimilarity matrix on square-root transformed SV’s was chosen for subsequent analysis in Primer-E v7. PERMANOVA was used to test whether the bacterial composition differed between communities, sample type (water vs biofilm) or chlorination status. Sample sites along the DWDS were included as random factor nested in community and chlorination status. A distance-based test for homogeneity of multivariate dispersions (PermDISP) was conducted to test for differences in data dispersion between sample groups. A canonical analysis of principal coordinates (CAP) was performed to assess the predictive ability of the microbiota for sample type and chlorination status. A distance linear model and distance-based redundancy analysis (dbRDA) were performed to associate abiotic factors (water physicochemical factors, nutrients and metals) with the bacterial composition. Model selection was based on the lowest AICc and a combination of forward and backward step elimination. A negative binomial model on the non-rarefied data was applied in Phyloseq (DESeq2 library in R)[38] to identify bacterial taxa whose abundance significantly differed between sample groups. The False Discovery Rate (FDR) method was used to account for multiple testing. To compare the occurrence of SVs across sample groups, the R package labdsv was used and those SVs considered which occurred at least twice in the sample group. The R package Vennerable was used to generate Venn diagrams. Multiple regressions on the log transformed bacterial DNA abundance and negative binomial models on taxa sequence counts and SV richness (based on rarefied dataset) were performed in Stata-14 (www.stata.com) with standard errors clustered for sites and model residual diagnostics conducted. A result was considered significant if P<0.05 unless otherwise stated.

### Accession numbers

Sixteen-s rRNA gene sequencing data were submitted to the European Nucleotide Archive (PRJEB29497, ERR2882159 to ERR2882214). Accession numbers of *B*. *pseudomallei* whole genome sequencing data are in S2 Table.

## Results

### Water physicochemistry

**Fig 1:**
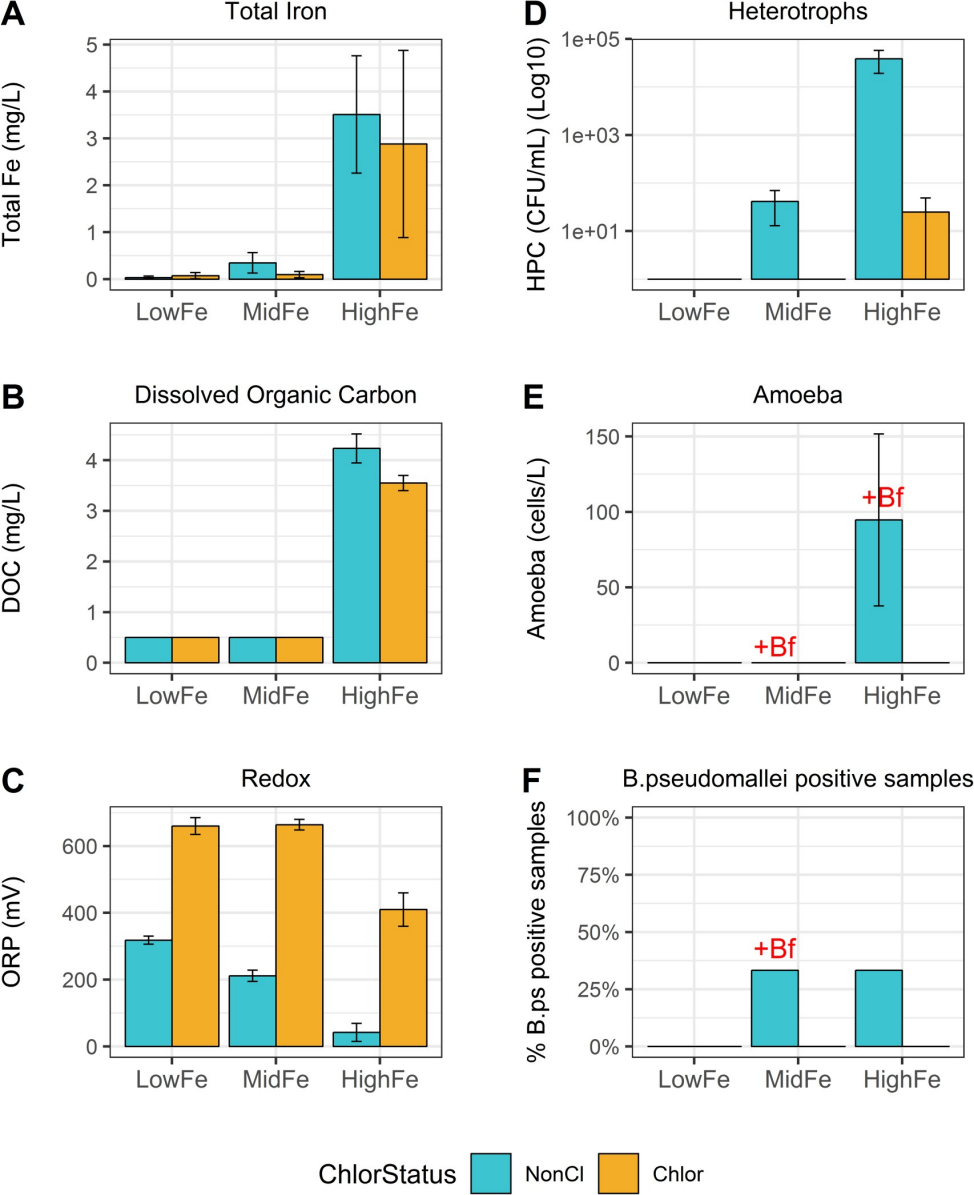
Physicochemical and culture data of water samples. For each water supply: n = 3 sites for unchlorinated and 2 sites for chlorinated parts of the supply–total 15 sites. “HighFe”, “MidFe” and “LowFe” mark the three remote water supplies. The colours signify the chlorination status (blue unchlorinated vs orange chlorinated). The bars show the mean and the error bars one standard error. Red “+Bf” mark culture positive biofilms at the corresponding sites. “HPC” Heterotrophic Plate Count, “CFU” Colony Forming Units. **A)** Total Iron; **B)** Dissolved Organic Carbon (DOC); **C)** Redox; **D**) Heterotrophic plate count; **E**) free-living amoebae; **F**) *B*. *pseudomallei* positive samples.

Water in the HF community had the highest levels of various metals, nutrients and salts (0.21–0.26 ppt) (Fig 1A–1C, S1 Table) and a neutral to slightly alkaline pH (6.9 to 7.8). The MF community water had lower iron levels of 0.03 to 0.78 mg/L, generally lower metal and nutrient levels and was more acidic (pH 4.8 to 5.3). Water in the LF community was the least buffered with the lowest metal and nutrient levels of the three water supplies and also the most acidic with a pH of below 5 for all five tested water samples (S1 Table). For all three communities, total Fe levels were strongly correlated with total Mn levels (Spearman’s rho 0.91, P<0.001). The DO and redox levels reflected the water origin with oxygen-deprived groundwater sampled from the bores showing the lowest DO and ORP levels. Redox levels overall were lowest at the HF community indicating a more reducing environment which was also reflected by the more neutral pH and higher DOC levels.

### Water distribution system microbiota

#### Microbial culture results

Fig 1D–1F show the culture results of heterotrophs, free-living amoebae and *B*. *pseudomallei* of water and biofilm samples. All samples were negative for *E*. *coli*. Water and biofilms from the unchlorinated tank and rising main in the HF community showed abundant microbial growth including coliforms, *P*. *aeruginosa*, *Hartmannella* amoebae and *Naegleria lovaniensis*. No heterotrophic microbes were recovered from the majority of chlorinated biofilm and water samples except one chlorinated sample from the HF community grew 49 CFU/mL heterotrophs. *Burkholderia* spp. bacteria (not *B*. *pseudomallei*) were cultured from a chlorinated water sample in the MF community. Water from a bore in the HF community and a bore in the MF community were positive for *B*. *pseudomallei*. The biofilm of the latter was also positive for *B*. *pseudomallei* and also contained *Hartmanella* amoebae.

#### *B*. *pseudomallei* WGS analysis

Six *B*. *pseudomallei* isolates from water and biofilms of the HF and MF water supply were whole genome sequenced. The six isolates consisted of five different MLST sequence types (Table 1); three of which (STs 113, 678 and 731) have previously been detected in the 1990’s in melioidosis patients from remote communities. Genomes of *B*. *pseudomallei* from the MF water supply contained the *Yersinia*-like fimbrial gene cluster (YLF) and the filamentous hemagglutinin gene, *fhaB3*. The lipopolysaccharide (LPS) O-antigen type B was found in *B*. *pseudomallei* isolated from the HF supply together with the *Burkholderia mallei*-like allele of the *bimA-*_*Bm*_ gene (*Burkholderia* intracellular motility A) involved in actin polymerization and intracellular motility.

**Table 1:** Geographical and virulence genetic markers of six *B*. *pseudomallei* isolates.

**Isolate**	**Water Supply**	**Sample type**	**MLST**	**YLF-BTFC**	**LPS**	***bimA***	***fhaB3***
MSHR10126	HF	Water	1591	BTFC	LPS B	*Bm*	Negative
MSHR10130	HF	Water	113	BTFC	LPS B	*Bm*	Negative
MSHR10274	MF	Biofilm	731	YLF	LPS A	*Bp*	Positive
MSHR10302	MF	Biofilm	731	YLF	LPS A	*Bp*	Positive
MSHR10283	MF	Biofilm	678	BTFC	LPS A	*Bp*	Negative
MSHR10275	MF	Water	1651	YLF	LPS A	*Bp*	Positive

*In silico* analysis of WGS of six *B*. *pseudomallei* isolated from the HF and MF water supply. YLF *Yersinia*-like fimbrial gene cluster; BTFC *Burkholderia thailandensis*-like flagella and chemotaxis cluster; LPS lipopolysaccharide; *bimA Burkholderia* intracellular motility A; *fhaB3* filamentous hemagglutinin gene

**Fig 2:**
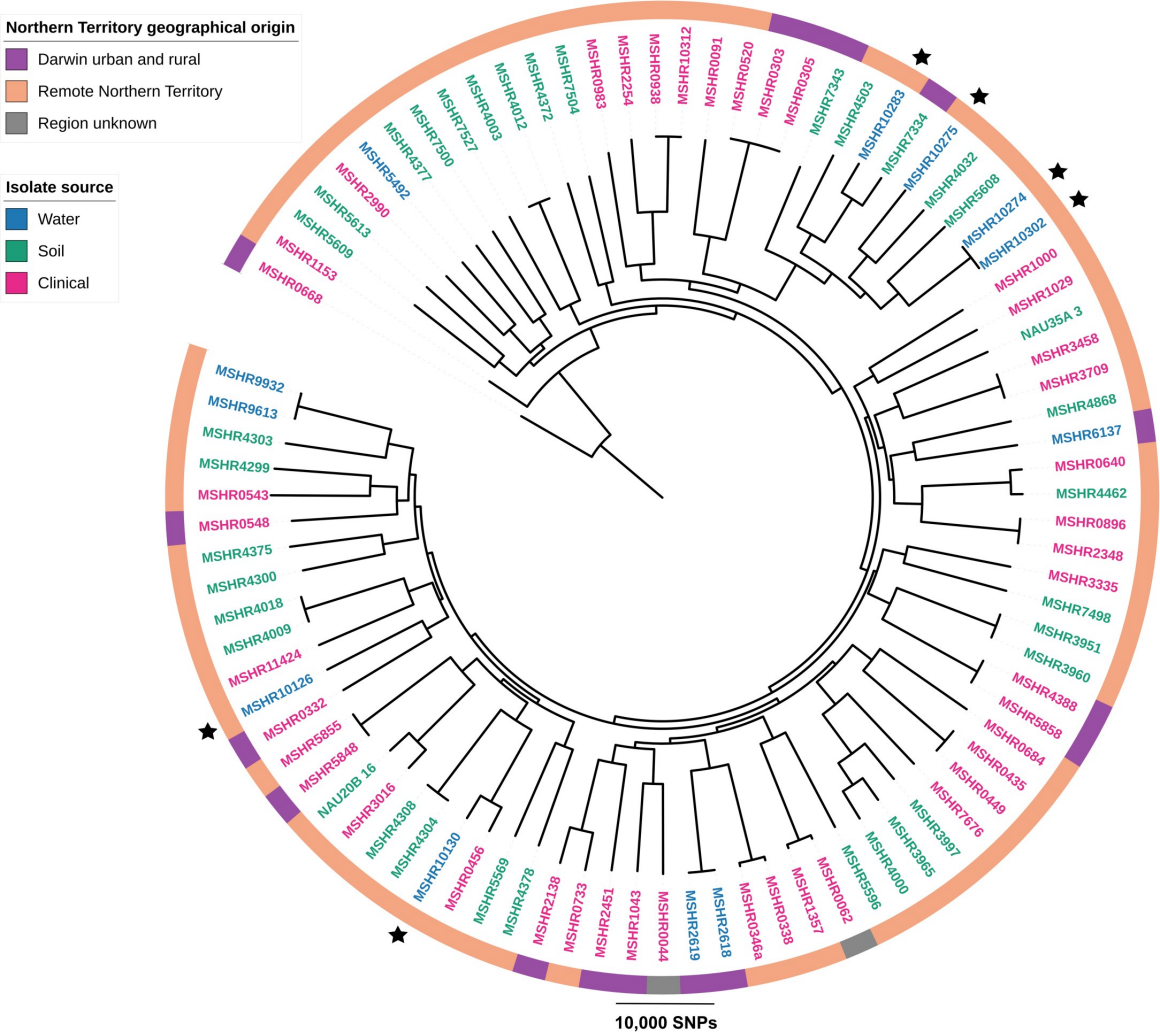
Phylogenetic tree of *B*. *pseudomallei* isolates from the Northern Territory. Maximum-parsimony phylogenetic tree of 89 *B*. *pseudomallei* isolates from the Northern Territory isolated from soil, water or of clinical origin. The tree is based on 174,905 orthologous core-genome SNPs with a consistency index of 0.2. All WGS are accessible on NCBI (S2 Table). Isolates marked with stars are isolates recovered in this study. See Table 1 for details.

A phylogenetic tree based on the WGS of these six and 83 other *B*. *pseudomallei* isolates of the NT showed a cluster of the MF water supply isolates with isolates from soil of other remote parts of the NT but also the Darwin region. One isolate from the HF water supply (MSHR10130) clustered closest with an isolate (MSHR0456) with the same ST113 from a remote patient from 1996 (Fig 2)(S2 Table). Despite sharing the same ST, these two isolates were still more than 5,000 orthologous SNPs apart.

#### The microbiota based on 16s rRNA gene qPCR and sequencing

**Fig 3:**
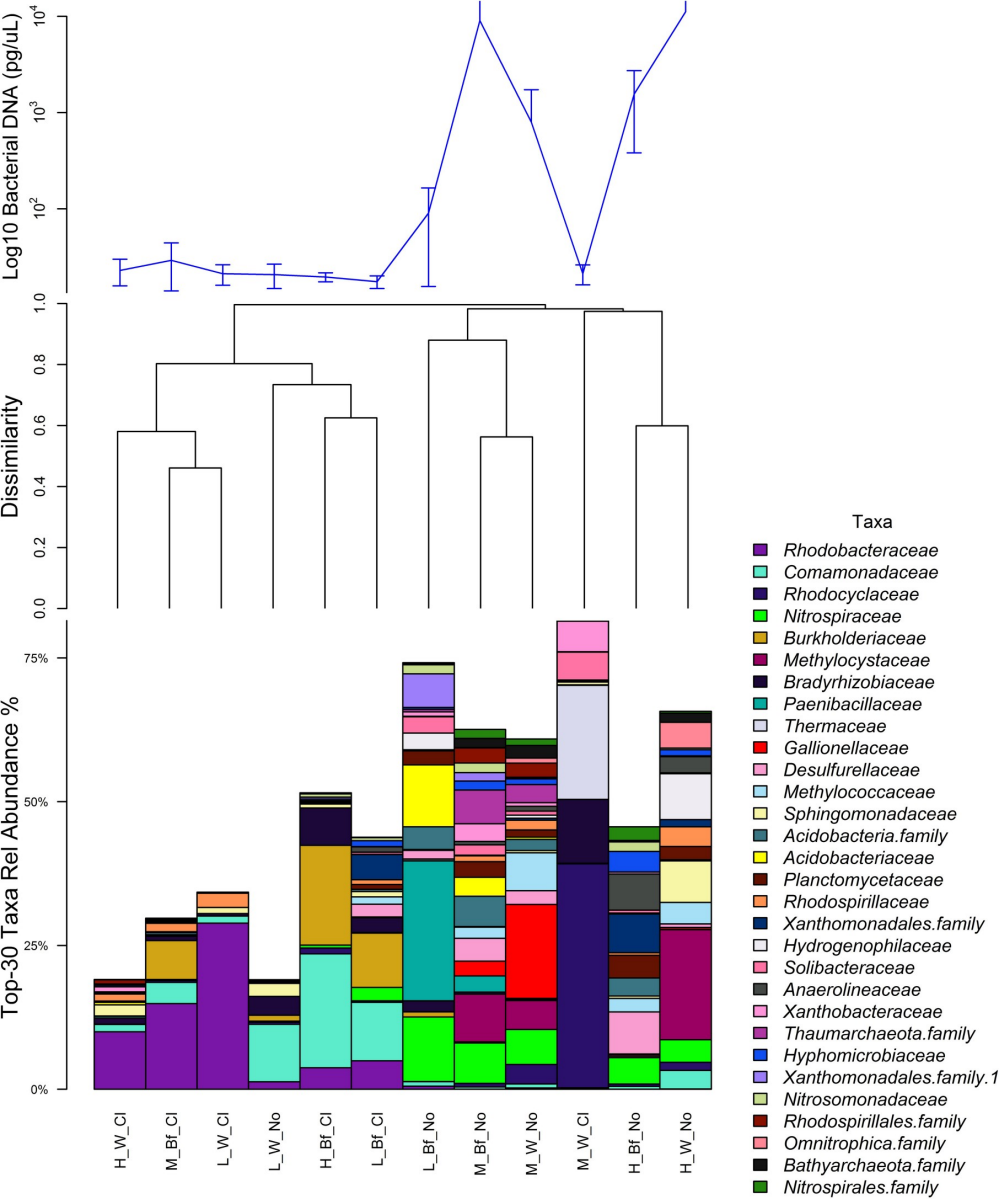
Bacterial relative abundance and composition in water supplies. **Top**: Bacterial relative abundance based on 16s rRNA gene qPCR—results grouped by water supply, sample type and chlorination status. Error bars mark one standard deviation. The y axis is in log-10 scale. The order of groups is according to the hierarchical cluster analysis (Mid). **Mid**: Hierarchical cluster analysis of the corresponding microbiota with rarefied SVs averaged per group of samples. **Bottom**: Bar plot of the 30 most abundant taxa at family level (where known). Group labels starting with H, M and L indicate the water supply i.e. HighFe, MidFe, LowFe, followed by W (Water) or Bf (Biofilm) and Cl (chlorinated or treated) vs No (untreated).

On average 19 times more bacterial DNA was found in the source water from MF (P = 0.002) and 83 times more in the HF water supply (P<0.001) compared to the source water of the LF water supply. Compared to the corresponding source water, bacterial DNA was considerably lower in disinfected parts of the water supplies HF (average 98.7% reduction, P<0.001) and MF (94.4% reduction, P = 0.005) but not LF (no difference, P = 0.97) (Fig 3 top). Microbial richness showed a similar trend as abundance. There were on average 3.9 times more sequence variants (SVs) in the MF source water (P<0.001) and 4.2 times more in HF (P<0.001) compared to the source water of LF (S2 Fig). While there was no difference in richness between bulk water and biofilms for the MF and HF water supply, for LF the biofilm richness was 79% higher compared to bulk water (P = 0.040). Water disinfection had the largest effect on richness for the MF water supply with a 91.4% reduction in SVs in water (P<0.001) followed by 76.3% (P<0.001) for HF and no detectable reduction for the LF water supply. The microbial richness in chlorinated biofilms was 2.9 times higher compared to chlorinated bulk water for the LF (P<0.001) and MF (P = 0.001) water supplies.

Proteobacteria were the most common phylum in the water supplies comprising more than 40% of 16s rRNA gene sequences for most samples. Alpha- and Betaproteobacteria were common in the untreated source samples while Gammaproteobacteria were more abundant in the chlorinated samples. There was a distinct relative increase of several microbial families in chlorinated water and biofilm compared to unchlorinated samples (Fig 3 bottom). These included *Comamonadaceae*, *Rhodobacteraceae* and *Burkholderiaceae*. A NCBI Blast analysis identified the latter as uncultured *Burkholderia* spp. or taxa belonging to the new genus *Paraburkholderia* [39]. For untreated parts of the DWDS, *Gallionellaceae* were abundant in water of MF, while HF contained more *Methylocystaceae*, *Anaerolineaceae* or *Sphingomonadaceae* (Fig 3 bottom). Most untreated samples contained nitrite oxidizing *Nitrospiraceae* with the highest relative abundance in untreated biofilms (15–35% relative abundance). These comprised the genera *Nitrospira*, *Leptospirillum* or uncultured bacteria. Uncultured bacteria of the family *Nitrosomonadaceae* were widespread in unchlorinated biofilms at low abundance (< 5%).

A cluster analysis and nMDS showed a large divide of microbiota samples according to chlorination status (Figs 2-mid and S3). The nMDS showed distinct clustering of the unchlorinated microbiota according to water supply with the HF samples differing most from the other water supplies (S3 Fig). The microbiota of water and biofilms clustered together, especially for the untreated MF water supply. For the LF water supply the composition of the unchlorinated microbiota was most similar to the chlorinated microbiota cluster. Once chlorinated, the microbial composition could no longer be distinguished between water supplies and clustering according to sample type was more apparent.

A PERMANOVA analysis on the microbial compositions confirmed the nMDS patterns showing a difference according to water supply (P = 0.002) and chlorination status (P = 0.001) but not between bulk water and biofilms overall (P = 0.16) (Table 2). A pairwise comparison revealed that the microbiota differed between sample types for chlorinated samples (P = 0.025) but not for untreated samples (P = 0.17). The untreated microbiota samples were more heterogeneous compared to their chlorinated counterparts (PermDISP P = 0.001; Table 2) and samples were also more heterogeneous in the MF and HF water supply compared to LF (PermDISP P = 0.001).

**Table 2:** PERMANOVA analysis.

Factor PERMANOVA	Pseudo-F (df)	ECV	P value	PermDISP P value
Chlorination (y/n)	2.4 (1)	22.8	0.001***	0.001***
Water Supply (HF/MF/LF)	1.7 (2)	19.7	0.002**	0.002**
Sample type (bf/w)	2.0 (1)	16.2	0.157	0.171
Sites (random factor)	5.2 (9)	34.1	0.001***	0.311
IA Sample type x Community	1.8 (2)	24.7	0.186	
IA Community x Chlorination	1.4 (2)	21.7	0.055	
IA Sample type x Chlorination	1.9 (1)	21.6	0.179	
IA Sites x Sample type	3.1 (3)	34.1	0.001***	

PERMANOVA analysis testing differences in the microbiota between sample type (water vs biofilm), water supply (HF, MF, LF), chlorination status (chlorinated vs non-chlorinated), random factor sites (# 15 sites) and their interactions (IA marked with an “x”). ^a^ “df” degrees of freedom, “ECV” square root of estimates of components of variation indicating the effect size as average % SV dissimilarity due to that factor (residual ECV 33.5). P value is based on >990 unique permutations.

*** P value = 0.001

** P value <0.01.

The majority of SVs (79%, n = 4,295) only occurred in untreated samples. Of those SVs, 37% were exclusively found in unchlorinated water, 30% in unchlorinated biofilms and 33% occurred in both (S4 Fig). In contrast, only 5% of SVs occurred exclusively in treated samples and only 1.7% of SVs (n = 90) occurred in all categories, untreated and treated water and biofilm samples.

**Fig 4:**
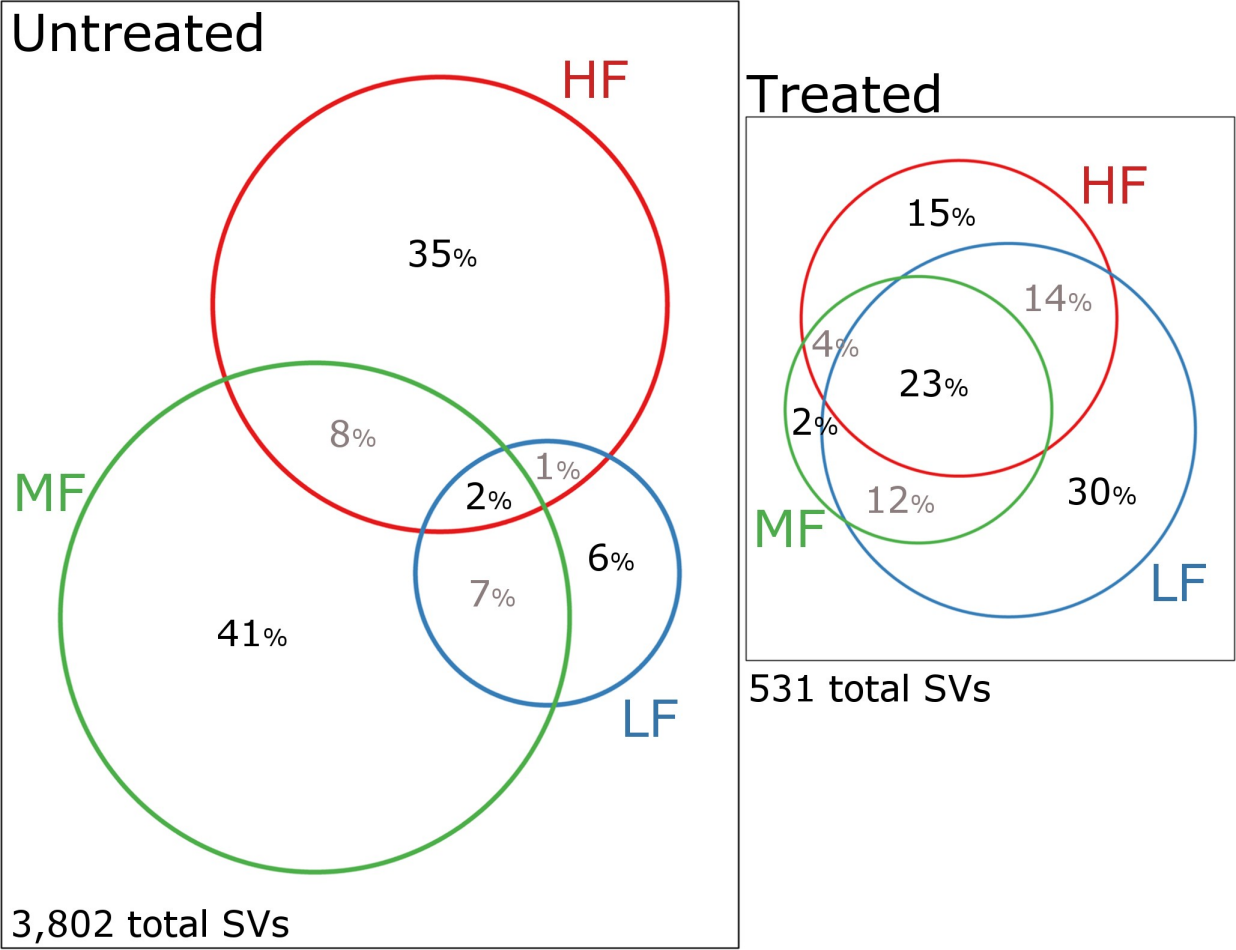
Venn diagram of SVs unique and shared between water supplies. Venn diagrams showing the percentage of SVs unique or shared between water supplies for untreated (left) and treated samples (right). Bulk water and biofilm samples were combined. Only SVs which occurred in minimum two samples were considered.

A comparison of SVs between water supplies showed that for untreated samples, 41% and 35% of SVs were exclusive to the MF and HF water supply with only 6% of SVs exclusive to the LF water supply and only 2% (n = 61) shared between all supplies (Fig 4). The opposite was the case for treated samples, where 30% of SVs only occurred in the LF treated samples, 23% occurred in all three supplies while only 2% and 15% were exclusive to the MF and HF supply (Fig 4).

#### The association of the microbiota with abiotic water factors

DO and ORP explained most (each 12%) of the microbial composition followed by free chlorine concentrations and TDN (each 9.8%)(all P = 0.001) (S3 Table). A strong association was found between the microbiota from the HF community and raised Fe, S, TDN and TDP levels (S5 Fig). The chlorinated microbiota were associated with higher redox and dissolved oxygen levels and microbiota from untreated MF samples with raised turbidity (S5 Fig).

#### The association of bacterial taxa with sample groups

**Fig 5:**
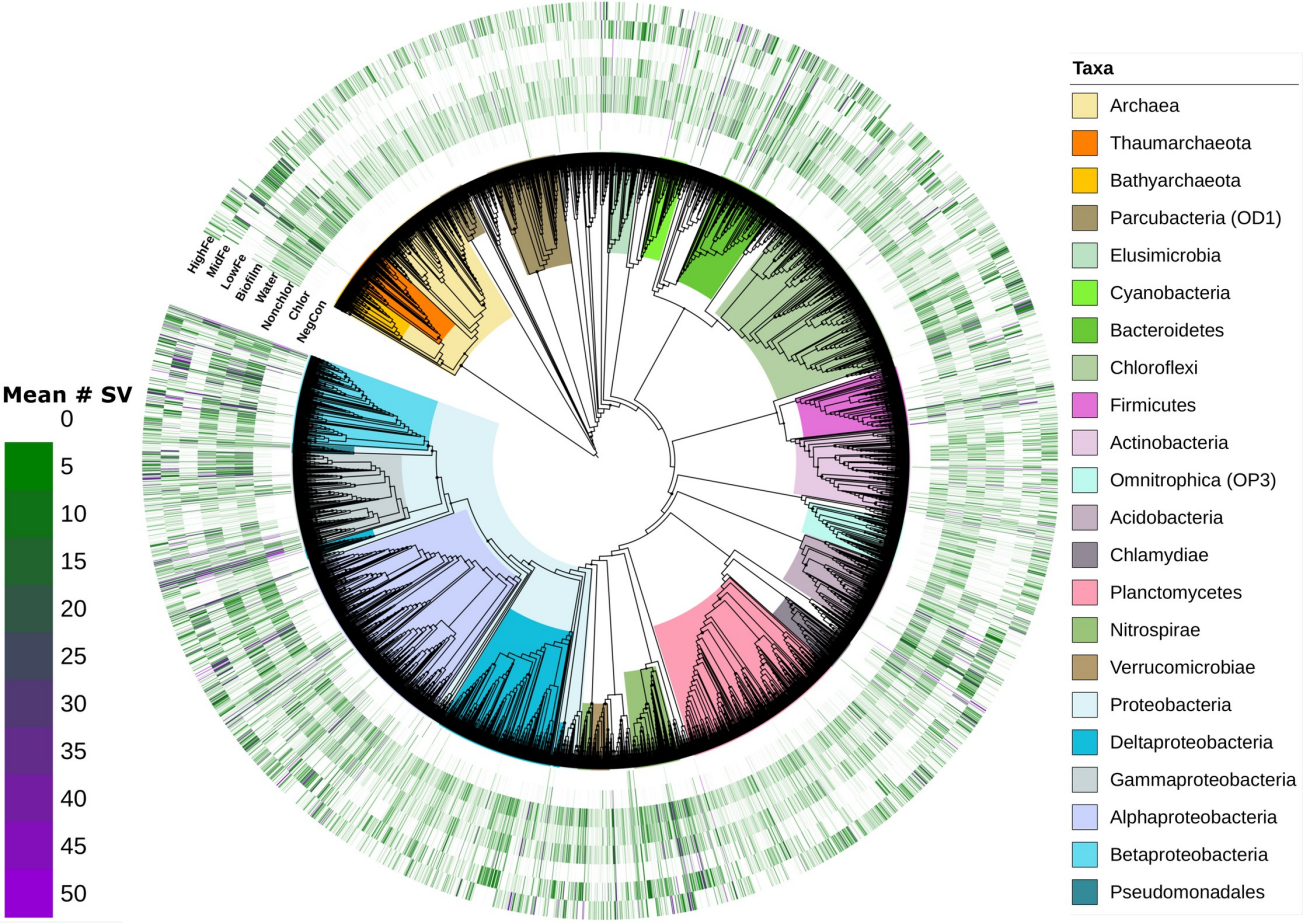
Phylogenetic tree of SVs and relative abundance in sample groups. Phylogenetic tree of 16s rRNA gene SVs of all samples including negative controls after exclusion of contaminant and rare SVs. The average SV abundance in different groups of samples (outer circles) is shown across different levels of taxa resolution (tree). The midpoint-rooted tree was generated in QIIME-2 using the FastTree-2 routine [40] on a multiple alignment using MAFFT (Multiple Alignment Fast Fourier Transform) and with gaps across all sequences removed. The tree and metadata were visualized in iTOL (https://itol.embl.de/).

Relative abundance of SV’s at different taxa resolutions showed a distinctively higher abundance of Thaumarchaeota in unchlorinated MF samples compared to HF and LF (Fig 5). Generally, water contained more SVs of Bathyarchaeota while biofilms had more *Acidobacteriaceae*. The inclusion of negative controls proved important for the analysis of chlorinated samples. Many SVs detected in chlorinated samples were also detected in negative controls and therefore, were not considered further (Fig 5). SVs of the phylum Actinobacteria including Acidimicrobiales were abundant in chlorinated samples and absent in negative controls.

All 16 untreated biofilm samples were classified correctly in a CAP analysis constrained for sample type and chlorination (S6 Fig) confirming a specific microbial fingerprint for these samples. SVs of the taxa Chloroflexi, Thaumarchaeota or *Planctomycetaceae* were correlated most with unchlorinated biofilms while *Gallionellaceae* were most abundant in unchlorinated water samples (Pearson correlation to CAP axes > 0.5 for all). SVs most correlated with chlorinated samples belonged to Gammaproteobacteria.

To further explore the association of SVs with sample groups, negative binomial models were conducted on the non-rarefied microbiota. SVs of *Burkholderiaceae*, *Nitrospiraceae* or *Nitrosomonadaceae* were more common in untreated biofilms compared to bulk water while SVs of *Comamonadacea* and Acidimicrobiales were more abundant in chlorinated biofilms compared to untreated biofilms. *Sphingomonadaceae* bacteria were more often found in samples which cultured amoebae and the microbiota of the water sample which grew 49 *Hartmanella* / L consisted of 11–21% *Sphingomonadaceae*. There was a significant association of *B*. *pseudomallei* culture positive samples with a SV of the genus *Nitrospira*. This genus had the highest relative abundance (14–15%) in the two *B*. *pseudomallei* positive biofilm replicates in the bore head of the MF community and was one of the top-10 genera for both *B*. *pseudomallei* positive water replicates of that bore. For the *B*. *pseudomallei* positive water sample of the HF water supply, uncultured *Nitrospiraceae* were the second most abundant taxa with 8% relative abundance.

#### Detection of taxa containing iron cycling bacteria

The iron oxidizing genus *Gallionella* was abundant in water of two of three tested bores of the MF community including the *B*. *pseudomallei* positive water and biofilm sample. It was absent in the samples tested from the LF and HF communities. In contrast, *Geobacter*, *Azospirillum* or *Ferrovibrio* were primarily found in the untreated parts of the HF community and most abundant in its biofilms. *Thiothrix*, *Thiobacillus* and *Desulfobulbus* involved indirectly in iron cycling were also primarily found in the bores and tank of the HF water supply while *Desulfovibrio* was also detected in the MF bores.

#### Detection of genera containing opportunistic human pathogens in chlorinated samples

Six *Mycobacteria* SVs were mainly found in chlorinated water and biofilm samples of all three water supplies (S7 Fig) while absent in all five negative controls. There was no negative correlation between these SV counts and the DNA concentration indicating that these SVs were not contaminants. One *Mycobacterium* SV was amongst the two most abundant SVs (13.2% and 6.9%) at one chlorinated site of the MF water supply. While *Pseudomonas* DNA was detected in chlorinated water and biofilm samples, it also occurred in 3/5 negative controls and thus, was not considered further.

## Discussion

In this study we analysed a snapshot of the microbiota in bulk water and biofilms in the source and distribution system of three remote communities in Northern Australia. Changes in the microbiota were associated most with changes in redox levels and dissolved oxygen followed by various metals and nutrients such as TDN or DOC. These parameters not only differed along the water treatment train but also between the water supplies. Indeed, the geochemistry of the groundwater varied considerably between the three water supplies which was also reflected by significant differences in the source water microbiota. Remarkably, only 2% of SVs in untreated source samples occurred in all three water supplies as opposed to 23% SVs shared between the supplies for the treated parts. Significant microbial differences in source water of water supplies driven by geochemical differences have previously been reported [41].

The water supply of community LF was fed by the deepest aquifer, had the newest constructed bores and the water contained the least amount of nutrients and was the most acidic. There were fewer and less diverse bacteria in the water and while biofilm growth in the pipes was minimal, this was the only water supply with a significantly higher richness in the untreated biofilms compared to untreated bulk water. The scarce biofilms of all three tested LF bores were rich in chemo-organotrophic *Acidobacteriaceae*. Bacteria from these taxa typically have a growth optimum at lower pH and have adapted strategies to grow in low-carbon environments [41–43].

There were significantly more metals, bacteria and archaea in the bore water of the MF water supply. The MF bore heads were covered in thick loose iron flocs and biofilms and contained abundant *Gallionella* bacteria [44–46]. Ideal conditions for *Gallionella* growth have been reported to be at a neutral to slightly acidic pH, with a redox potential of 200–320 mV [47] matching conditions in the MF water supply. The bore field of the MF community is often inundated during the wet season during which contamination with surface water is possible. Two of the three tested bores were fed by a shallow aquifer and water from these bores grew heterotrophic bacteria. In contrast, the third bore accessed the Kombolgie sandstone aquifer at 20 meters depth, only had scarce heterotrophic growth but its water and biofilm samples were positive for *B*. *pseudomallei*. It is not known whether *B*. *pseudomallei* indeed occurred in the deeper aquifer. *B*. *pseudomallei* is a facultative anaerobe and hardy bacterium able to survive even in distilled water [16, 48]. Alternatively, the bore could have been contaminated with surface water during the wet season although the scarce heterotrophic growth did not support the notion of a recent contamination. Previous research has shown that *B*. *pseudomallei* inhabits shallow unconfined aquifers [49–51] and *B*. *pseudomallei* has been found more often in residential bores with hard water, acidic pH, increased iron levels and turbid water also containing coliforms [14]. These are indicators for surface water influx or water from shallow seasonal inter-flow aquifers. More research is needed to establish the potential occurrence of *B*. *pseudomallei* also in deeper aquifers which would be more difficult to manage by water providers.

Free-living *Hartmannella* amoebae were also recovered from the *B*. *pseudomallei* positive biofilm. Similar to other opportunistic pathogens, *B*. *pseudomallei* is able to survive within amoebae as shown in laboratory experiments [52, 53]. Survival within amoebae increases the pathogens’ resistance to chlorination [53].

Community HF was built in a coastal swamp area with shallow unconfined aquifers. Accordingly, the groundwater was buffered with the highest levels of various nutrients and metals. Untreated samples had the largest microbial richness and the pipes were covered in biofilms and iron deposits of a firm and scaly nature. Water from HF had a lower redox potential indicating a more reducing environment and organic carbon levels were high. Consequently, despite the high iron levels no *Gallionella* were recovered but instead dissimilatory iron reducers or nitrate-dependent anaerobic iron oxidizers like *Geobacter*, *Azospirillum* or *Ferrovibrio* [54]. The sulphur oxidizing *Thiobacillus* or *Thiothrix* were also detected. Bacteria of these genera cause biogenic sulphuric acid corrosion of concrete and they produce sulphates used by sulphate reducing bacteria such as *Desulfovibrio* or *Desulfobulbus*, both of which were also found at HF and less so at MF [45]. Sulphate reducing bacteria are involved in anaerobic corrosion or pitting of iron or steel by producing hydrogen sulphide and promoting anaerobic iron oxidation [55]. The untreated tank and rising main of the HF water supply showed abundant microbial life which flourished in the warm nutrient-rich water with high heterotroph counts, coliforms and *Hartmannella* and *Naegleria lovaniensis* amoebae feeding on the bacteria. It was of interest that these samples were also rich in *Sphingomonadaceae* bacteria which have been identified as an abundant member of the intra-amoebal microbiota in drinking water [56]. They have also been described in biofilms of chlorinated parts of water supplies and may be a reservoir of antibiotic resistant genes [57]. Water and biofilms of the tank also grew *P*. *aeruginosa*, an opportunistic pathogen primarily known for its pathogenicity in nosocomial settings and potential spread of antibiotic resistant genes in water distribution systems [58, 59]. In contrast to *P*. *aeruginosa*, there were no *B*. *pseudomallei* detected in the tank. Instead, *B*. *pseudomallei* was cultured from the shallow HF bore. Similar to the *B*. *pseudomallei* positive bore at MF, this bore only had scarce heterotrophic growth. Heterotrophic microbes require organic carbon for growth and HPC are routinely used by water providers to monitor the integrity of the supply and to indicate surface water contamination or presence of biofilms [22]. In this study, increased HPC did not match the presence of *B*. *pseudomallei*.

Genome analysis of the *B*. *pseudomallei* isolates revealed the presence of the YLF gene cluster and *fhaB3* gene in isolates from the MF bore. The YLF cluster is more common in *B*. *pseudomallei* from Southeast Asia and remote parts of the Northern Territory [60, 61] while *fhaB3* has been associated with *B*. *pseudomallei* positive blood culture as opposed to localized skin lesions [61]. LPS type B was found in *B*. *pseudomallei* from the HF supply together with the *bimA-*_*Bm*_ gene. Both these genetic markers are more common in *B*. *pseudomalllei* from remote NT and *bimA-*_*Bm*_ is also more widespread in Southeast Asia [62]. The *bimA-*_*Bm*_ gene has been associated with neurological disease [61]. A phylogenetic tree with the water supply and other NT isolates showed no closely related *B*. *pseudomallei* isolates of clinical origin.

Nitrifying *Nitrospiraceae* were abundant in the untreated biofilms. Their production of nitrates provides a source of nutrients increasing biofilm mass [63]. Most nitrifiers identified in this study belonged to the genus *Nitrospira* common in drinking water with a preference for low nutrient or low nitrite environments [41, 64]. It was of interest that nitrate producing *Nitrospiraceae* were associated with *B*. *pseudomallei* positive samples. *B*. *pseudomallei* is a denitrifier under anaerobic conditions and in one study, *B*. *pseudomallei* load increased in sand upon nitrate treatment while in another study, *B*. *pseudomallei* was associated with soil containing elevated total nitrogen [65, 66]. More research is needed to further explore this potential commensal relationship.

Chlorination successfully contained *B*. *pseudomallei* and *P*. *aeruginosa* and reduced nuisance organisms. Similar to other studies, water treatment had the largest impact on the microbiota [67, 68]. The largest reduction in bacterial richness was observed for the MF water supply. Water disinfection of this water supply also included UV treatment apart from chlorine gas. Gammaproteobacteria were more abundant in chlorinated samples across all water supplies and members of this taxa are more resilient to higher chlorine levels and oxidative stress compared to Alpha- and Betaproteobacteria [69, 70]. One chlorinated site of the MF water supply had abundant DNA of several sequence variants of another group of opportunistic pathogens, called non-tuberculous mycobacteria. Further investigations are needed to establish whether these were from viable bacteria. Environmental mycobacteria are known to persist in water supplies and can cause disease in immunocompromised people or people with chronic lung disease [71].

Due to the low biomass of many samples in this study, the inclusion of several negative controls proved crucial. Various sequence variants in chlorinated samples were also detected in negative controls such as those of *Ralstonia* or *Pseudomonas*. This made it difficult to differentiate between hardy bacteria persisting in various environments including chlorinated water or mere contaminants of laboratory reagents and DNA extraction kits [36, 72]. As outlined in the methods, utmost care was taken in excluding samples with low sequence numbers and/or similarity to microbial fingerprints of negative controls and excluding potential contaminant sequence variants. Subsequent studies will use larger water sample volumes and filters with smaller pore size to increase biomass and ensure capturing microbes of all sizes [70].

Overall, there were no significant differences in the microbiota between bulk water and biofilms; this was particularly the case for the turbid water of the MF supply with a high level of suspended solids. Swabs were used to collect biofilms which primarily captured the top layer of biofilms or microbes associated with suspended solids and loose deposits as opposed to other studies which scraped the biofilm off pipes or grew them on coupons inserted into pipes [73]. Nevertheless, we found untreated biofilms to be more heterogeneous than planktonic microbiota with a distinct microbial fingerprint for each water supply. Sequence variants of various nitrifying families were more common in untreated biofilms compared to untreated bulk water as previously reported [74]. Once the water was treated, the microbiota indeed differed between water and biofilms and the proportion of SVs unique to biofilms also increased while the proportion of SVs shared between the sample types decreased. This matches previous reports of an increase in differences between sample types upon water treatment [67].

In summary, we found that the geochemistry of the source water had a substantial impact on the untreated microbiota with largely different microbial communities in untreated parts of the three water supplies. Accordingly, a multiple barrier approach to improve water quality would have to account for the heterogeneous nature of the microbiota in different water supplies across Northern Australia. We detected three opportunistic pathogen groups; namely non-tuberculous mycobacteria, *P*. *aeruginosa* and *B*. *pseudomallei*. In contrast to our working hypothesis, *B*. *pseudomallei* was cultured from a bore accessing a deeper aquifer and future investigations across seasons will determine whether *B*. *pseudomallei* indeed occurs in deeper confined aquifers or is mainly linked to surface or shallow aquifer water intrusions during the wet season, with the latter easier to manage for a water provider. Similar to other opportunistic pathogens in water supplies [20], *B*. *pseudomallei* was cultured from bulk water with low organic carbon and scarce heterotrophic growth. This matches its ability to thrive under nutritionally poor conditions [16, 48] but also indicates that HPC routinely used by water providers to monitor the supply integrity is a poor indicator for *B*. *pseudomallei* presence. We also detected *B*. *pseudomallei* in a multi-species biofilm linked to iron bacteria. Further research is needed to examine these interactions as *Gallionella* is increasingly used in biological iron-removal filters. This study provided a first snapshot of the microbiota in a selection of remote water supplies informing future studies to ultimately improve management guidelines for water supplies in the wet-dry tropics.

## Supporting information

S1 Table Physicochemical data, nutrient and metal levels of water samples. (PDF)

S2 Table Accession numbers and references of 89 *B*. *pseudomallei* WGS in phylogenetic tree. (PDF)

S3 Table Marginal tests of the distance linear model. (PDF)

S1 Fig Rarefaction curves of 16s rRNA gene sequences. (PDF)

S2 Fig Microbial richness of water and biofilm samples. (PDF)

S3 Fig nMDS of microbiota in water supplies. (PDF)

S4 Fig Venn diagram with number of SVs shared between sample types. (PDF)

S5 Fig dbRDA showing the association between the microbiota and abiotic factors. (PDF)

S6 Fig CAP analysis on the microbiota constrained by sample type and chlorination status. (PDF)

S7 Fig Mycobacteria SV counts across sample groups. (PDF)
