# Supplementary material for: Opportunistic pathogens and large microbial diversity detected in source-to-distribution drinking water of three remote communities in Northern Australia
Source: PLoS Negl Trop Dis. 2019 Sep 5;13(9):e0007672. doi: 10.1371/journal.pntd.0007672 (PMC6728021; doi:10.1371/journal.pntd.0007672)
Supplement: S1 Table — (PDF) [file pntd.0007672.s001.pdf]

S1 Table:

Water Supply		Low-Fe					Mid-Fe					High-Fe				
**Site**		**Bore 10**	**Bore 12**	**Bore 11**	**Retic 1**	**Retic 2**	**Bore 8**	**Bore 7**	**Bore 3**	**Retic 1**	**Retic 2**	**Tank**	**Bore**	**Rising Main**	**Retic 1**	**Retic 2**
**Phys-chem**	Free Cl mg/L	0	0	0	0.61	0.81	0	0	0	1.08	0.92	0	0	0	1.65	1.11
	Sal ppt	0.01	0.02	0.02	0.01	0.01	0.01	0.01	0	0	0.01	0.21	0.26	0.21	0.22	0.22
	pH	4.8	4.5	4.5	4.8	4.6	5.3	4.9	4.8	5.3	5.3	7.6	6.9	7.5	7.7	7.8
	DO %	88.7	74	72	87.9	92.6	45	21.5	33.5	90.1	91.6	65.7	24	59	85	90
	ORP mV	295	325	335	635	685	199	191	245	648	680	82	-9	54	360	460
**Nutrients (mg/L)**	TDN	0.03	0.04	0.03	0.05	0.05	0.04	0.06	0.11	0.11	0.22	0.35	0.29	0.35	0.14	0.12
	NOx	0.024	0.035	0.025	0.044	0.046	0.028	0.035	0.092	0.1	0.1	0.049	<0.002	0.081	0.061	0.055
	TDP	0.022	0.022	0.024	0.022	0.023	0.065	0.030	0.024	0.020	0.020	0.034	0.036	0.034	0.030	0.030
	DOC	<0.5	<0.5	<0.5	<0.5	<0.5	<0.5	<0.5	<0.5	<0.5	<0.5	4	4.8	3.9	3.7	3.4

Water Supply		Low-Fe					Mid-Fe					High-Fe				
**Site**		**Bore 10**	**Bore 12**	**Bore 11**	**Retic 1**	**Retic 2**	**Bore 8**	**Bore 7**	**Bore 3**	**Retic 1**	**Retic 2**	**Tank**	**Bore**	**Rising Main**	**Retic 1**	**Retic 2**
**Elements (ug/L)**	Fe	97	0.9	0.6	139	7	127	783	131	30	163	1,014	4,607	4,913	4,879	886
	Mn	0.4	0.2	0.4	0.9	0.2	3.3	7.4	19.6	2.1	1.1	337	471	3,051	793	133
	Mg	230	264	321	280	282	1,275	380	535	342	355	11,325	6,787	11,194	11,247	11,241
	K	35	54	103	40	40	375	193	144	148	154	2,530	1,464	2,553	2,534	2,542
	Ca	23	35	58	677	38	384	195	297	893	752	63,121	84,430	63,614	63,115	63,232
	S	<	<	<	<	<	<	<	<	<	<	3,495	9,893	3,490	3,509	3,521
	Mo	<	<	<	<	<	<	<	<	<	<	0.8	0.5	0.5	0.5	0.5
	Ni	0.0	0.0	0.2	0.1	0.0	0.2	0.4	0.8	3.4	0.3	1.1	0.0	0.1	0.5	0.1
	Cu	0.0	17	19	41	33	5.8	4.6	5.3	319	627	0.2	1.0	0.8	0.2	1.1
	Zn	0.2	1.6	6.4	12	5.8	2.3	7.6	6.1	125	7.0	0.8	0.1	3.7	14	3.0

S1 Table Legend: Physicochemical data, nutrient and metal levels of water samples. “Free Cl” free chlorine levels in mg/L; “Sal” Salinity in ppt; ORP oxidation redox potential; Units of nutrients: mg N, P or C /L; Units of metals: µg/L; “Retic” chlorinated reticulation network
