# Supplementary material for: Opportunistic pathogens and large microbial diversity detected in source-to-distribution drinking water of three remote communities in Northern Australia
Source: PLoS Negl Trop Dis. 2019 Sep 5;13(9):e0007672. doi: 10.1371/journal.pntd.0007672 (PMC6728021; doi:10.1371/journal.pntd.0007672)
Supplement: S2 Table — (PDF) [file pntd.0007672.s002.pdf]

S2 Table:

B. ps isolate	Source	LPS type	Multi-locus ST	GenBank accession number	Genome Reference
MSHR0044	Clinical	A	882	JQIM00000000	Johnson et al., Genome Announc 2015 3(1):e01282-14
MSHR0062	Clinical	A	259	CP009234, CP009235	Johnson et al., Genome Announc 2015 3(1):e01282-14
MSHR0091	Clinical	B2	331	SUB5359248	BioRxv //dx.doi.org/10.1101/603886
MSHR0303	Clinical	A	36	JQDD00000000	Johnson et al., Genome Announc 2015 3(1):e01282-14
MSHR0305	Clinical	A	36	CP006469, CP006470	Stone et al., Genome Announc 2013 1(4):e00656-13
MSHR0332	Clinical	A	106	JQFM00000000	Johnson et al., Genome Announc 2015 3(1):e01282-14
MSHR0338	Clinical	A	243	ATJY00000000	Price et al., J Clin Microbiol 2015 53(1):282-86
MSHR0346a	Clinical	A	243	CP008763, CP008764	Daligault et al., Genome Announc 2014 2(6):e01106-14
MSHR0435	Clinical	A	126	JRFP00000000	Johnson et al., Genome Announc 2015 3(1):e01282-14
MSHR0449	Clinical	A	126	JQFO00000000	Johnson et al., Genome Announc 2015 3(1):e01282-14
MSHR0456	Clinical	B	113	JQFN00000000	Johnson et al., Genome Announc 2015 3(1):e01282-14
MSHR0520	Clinical	A	36	CP004368, CP004369	Johnson et al., Genome Announc 2015 3(2):e00159-15
MSHR0543	Clinical	B	294	JPZX00000000	Johnson et al., Genome Announc 2015 3(1):e01282-14
MSHR0548	Clinical	B	468	SUB5359248	BioRxv //dx.doi.org/10.1101/603886
MSHR0640	Clinical	A	109	JQFP00000000	Johnson et al., Genome Announc 2015 3(1):e01282-14
MSHR0668	Clinical	A	129	NC_009074, NC_009075	Johnson et al., Genome Announc 2015 3(2):e00159-15
MSHR0684	Clinical	A	103	JQDC00000000	Johnson et al., Genome Announc 2015 3(1):e01282-14
MSHR0733	Clinical	A	278	JQEE00000000	Johnson et al., Genome Announc 2015 3(1):e01282-14
MSHR0938	Clinical	B2	737	SUB5359248	BioRxv //dx.doi.org/10.1101/603886
MSHR0983	Clinical	A	142	JQDI00000000	Johnson et al., Genome Announc 2015 3(1):e01282-14
MSHR1000	Clinical	A	555	JQEF00000000	Johnson et al., Genome Announc 2015 3(1):e01282-14
MSHR10126	Environ	B	1591	SRR9333952	This paper

MSHR10130	Environ	B	113	SRR9333951	This paper
MSHR10274	Environ	A	731	SRR9333954	This paper
MSHR10275	Environ	A	1651	SRR9333953	This paper
MSHR10283	Environ	A	678	SRR9333956	This paper
MSHR1029	Clinical	A	145	JQDB00000000	Johnson et al., Genome Announc 2015 3(1):e01282-14
MSHR10302	Environ	A	731	SRR9333955	This paper
MSHR10312	Clinical	B2	737	SUB5359248	BioRxv //dx.doi.org/10.1101/603886
MSHR1043	Clinical	A	131	AOGU00000000	Price et al., mBio 2013 4(4):e00388-13
MSHR11424	Clinical	A	807	SUB5359248	BioRxv //dx.doi.org/10.1101/603886
MSHR1153	Clinical	A	117	CP009271, CP009272	Johnson et al., Genome Announc 2015 3(1):e01282-14
MSHR1357	Clinical	A	259	JQDA00000000	Johnson et al., Genome Announc 2015 3(1):e01282-14
MSHR2138	Clinical	A	456	JRFM00000000	Johnson et al., Genome Announc 2015 3(1):e01282-14
MSHR2254	Clinical	B2	770	SUB5359248	BioRxv //dx.doi.org/10.1101/603886
MSHR2348	Clinical	A	118	SUB5359248	BioRxv //dx.doi.org/10.1101/603886
MSHR2451	Clinical	A	483	JQEG00000000	Johnson et al., Genome Announc 2015 3(1):e01282-14
MSHR2618	Environ	A	1485	SUB5359248	BioRxv //dx.doi.org/10.1101/603886
MSHR2619	Environ	A	1485	SUB5359248	BioRxv //dx.doi.org/10.1101/603886
MSHR2990	Clinical	A	781	JQHV00000000	Johnson et al., Genome Announc 2015 3(1):e01282-14
MSHR3016	Clinical	A	617	JQEH00000000	Johnson et al., Genome Announc 2015 3(1):e01282-14
MSHR3335	Clinical	B	901	JRFL00000000	Johnson et al., Genome Announc 2015 3(1):e01282-14
MSHR3458	Clinical	A	132	JQOB00000000	Johnson et al., Genome Announc 2015 3(1):e01282-14
MSHR3709	Clinical	A	132	JRFK00000000	Johnson et al., Genome Announc 2015 3(1):e01282-14
MSHR3951	Environ	A	1016	JPVA00000000	Johnson et al., Genome Announc 2015 3(1):e01282-14
MSHR3960	Environ	A	1016	JPVJ00000000	Johnson et al., Genome Announc 2015 3(1):e01282-14
MSHR3965	Environ	A	846	CP009152, CP009153	Johnson et al., Genome Announc 2015 3(1):e01282-14
MSHR3997	Environ	A	848	JQII00000000	Johnson et al., Genome Announc 2015 3(1):e01282-14
MSHR4000	Environ	A	846	JPVL00000000	Johnson et al., Genome Announc 2015 3(1):e01282-14

MSHR4003	Environ	A	849	JPUZ00000000	Johnson et al., Genome Announc 2015 3(1):e01282-14
MSHR4009	Environ	A	850	JQIL00000000	Johnson et al., Genome Announc 2015 3(1):e01282-14
MSHR4012	Environ	A	849	JPVH00000000	Johnson et al., Genome Announc 2015 3(1):e01282-14
MSHR4018	Environ	A	850	JQIK00000000	Johnson et al., Genome Announc 2015 3(1):e01282-14
MSHR4032	Environ	A	238	JPQL00000000	Johnson et al., Genome Announc 2015 3(1):e01282-14
MSHR4299	Environ	B	468	JPVC00000000	Johnson et al., Genome Announc 2015 3(1):e01282-14
MSHR4300	Environ	A	149	JPQI00000000	Johnson et al., Genome Announc 2015 3(1):e01282-14
MSHR4303	Environ	B	1019	JPVM00000000	Johnson et al., Genome Announc 2015 3(1):e01282-14
MSHR4304	Environ	A	995	JPOA00000000	Johnson et al., Genome Announc 2015 3(1):e01282-14
MSHR4308	Environ	A	995	JPVB00000000	Johnson et al., Genome Announc 2015 3(1):e01282-14
MSHR4372	Environ	A	877	JPQJ00000000	Johnson et al., Genome Announc 2015 3(1):e01282-14
MSHR4375	Environ	A	878	JPVI00000000	Johnson et al., Genome Announc 2015 3(1):e01282-14
MSHR4377	Environ	A	1020	JPQH00000000	Johnson et al., Genome Announc 2015 3(1):e01282-14
MSHR4378	Environ	A	1025	JQDP00000000	Johnson et al., Genome Announc 2015 3(1):e01282-14
MSHR4388	Clinical	A	562	SRR2887038	Currie et al., Emerging Infect. Dis. in press 2015
MSHR4462	Environ	A	109	JPQM00000000	Johnson et al., Genome Announc 2015 3(1):e01282-14
MSHR4503	Environ	A	1022	JPQN00000000	Johnson et al., Genome Announc 2015 3(1):e01282-14
MSHR4868	Environ	A	1026	JQGZ00000000	Johnson et al., Genome Announc 2015 3(1):e01282-14
MSHR5492	Environ	A	862	JQDO00000000	Johnson et al., Genome Announc 2015 3(1):e01282-14
MSHR5569	Environ	A	885	JQDL00000000	Johnson et al., Genome Announc 2015 3(1):e01282-14
MSHR5596	Environ	B	1027	JQDE00000000	Johnson et al., Genome Announc 2015 3(1):e01282-14
MSHR5608	Environ	B	1028	JPWQ00000000	Johnson et al., Genome Announc 2015 3(1):e01282-14
MSHR5609	Environ	A	1029	JQDJ00000000	Johnson et al., Genome Announc 2015 3(1):e01282-14
MSHR5613	Environ	A	866	JQDK00000000	Johnson et al., Genome Announc 2015 3(1):e01282-14
MSHR5848	Clinical	A	553	CP008909, CP008910	Daligault et al., Genome Announc 2014 2(6):e01106-14

MSHR5855	Clinical	A	553	CP008784, CP008783	Daligault et al., Genome Announc 2014 2(6):e01106-14
MSHR5858	Clinical	A	562	CP008891, CP008892	Daligault et al., Genome Announc 2014 2(6):e01106-14
MSHR6137	Environ	A	325	AXDS00000000	McRobb et al., J Clin Microbiol 2015 53(4):1144-48
MSHR7334	Environ	A	970	JQDF00000000	Johnson et al., Genome Announc 2015 3(1):e01282-14
MSHR7343	Environ	A	1030	JQDM00000000	Johnson et al., Genome Announc 2015 3(1):e01282-14
MSHR7498	Environ	A	766	JQDH00000000	Johnson et al., Genome Announc 2015 3(1):e01282-14
MSHR7500	Environ	A	1031	JREN00000000	Johnson et al., Genome Announc 2015 3(1):e01282-14
MSHR7504	Environ	A	1032	JPWR00000000	Johnson et al., Genome Announc 2015 3(1):e01282-14
MSHR7527	Environ	A	1033	JPWS00000000	Johnson et al., Genome Announc 2015 3(1):e01282-14
MSHR7676	Clinical	A	807	SUB5359248	BioRxv //dx.doi.org/10.1101/603886
MSHR896	Clinical	A	118	SUB5359248	BioRxv //dx.doi.org/10.1101/603886
MSHR9180	Clinical	B2	770	SUB5359248	BioRxv //dx.doi.org/10.1101/603886
MSHR9613	Environ	B	734	SUB5359248	BioRxv //dx.doi.org/10.1101/603886
MSHR9932	Environ	B	734	SUB5359248	BioRxv //dx.doi.org/10.1101/603886
NAU20B-16	Environ	A	617	CP004003, CP004004	Johnson et al., Genome Announc 2015 3(2):e00159-15
NAU35A-3	Environ	A	326	CP004377, CP004378	Johnson et al., Genome Announc 2015 3(2):e00159-15

S2 Table Legend: Accession numbers and references of 89 *B. pseudomallei* WGS from the Northern Territory, Australia. “Environ” environmental.
