# Supplementary material for: Opportunistic pathogens and large microbial diversity detected in source-to-distribution drinking water of three remote communities in Northern Australia
Source: PLoS Negl Trop Dis. 2019 Sep 5;13(9):e0007672. doi: 10.1371/journal.pntd.0007672 (PMC6728021; doi:10.1371/journal.pntd.0007672)
Supplement: S3 Table — (PDF) [file pntd.0007672.s003.pdf]

S3 Table:

Abiotic factor	Pseudo-F	P value	Proportion explained
DO	5.5	0.001	12.3%
ORP	5.3	0.001	12.1%
Cl_free	4.2	0.001	9.8%
TDN	4.2	0.001	9.8%
Mo	3.7	0.001	8.7%
DOC	3.4	0.001	8.0%
Mn	3.3	0.001	7.8%
Mg	3.2	0.001	7.6%
Ca	3.2	0.001	7.6%
K	3.2	0.001	7.5%
pH	3.1	0.001	7.4%
Sal	3.1	0.001	7.3%
TDP	3.1	0.001	7.3%
Fe	2.8	0.001	6.8%
S	2.7	0.001	6.5%
Turb	2.3	0.002	5.6%
Temp	2.0	0.011	4.8%
NO_x	1.9	0.017	4.7%
Ni	*1.0*	*0.457*	*2.4%*

S3 Table Legend: Marginal tests of the distance linear model showing the association between the microbial composition of the DWDS and abiotic factors.
