# Supplementary material for: Opportunistic pathogens and large microbial diversity detected in source-to-distribution drinking water of three remote communities in Northern Australia
Source: PLoS Negl Trop Dis. 2019 Sep 5;13(9):e0007672. doi: 10.1371/journal.pntd.0007672 (PMC6728021; doi:10.1371/journal.pntd.0007672)
Supplement: S1 Fig — (PDF) [file pntd.0007672.s004.pdf]

S1 Figure:

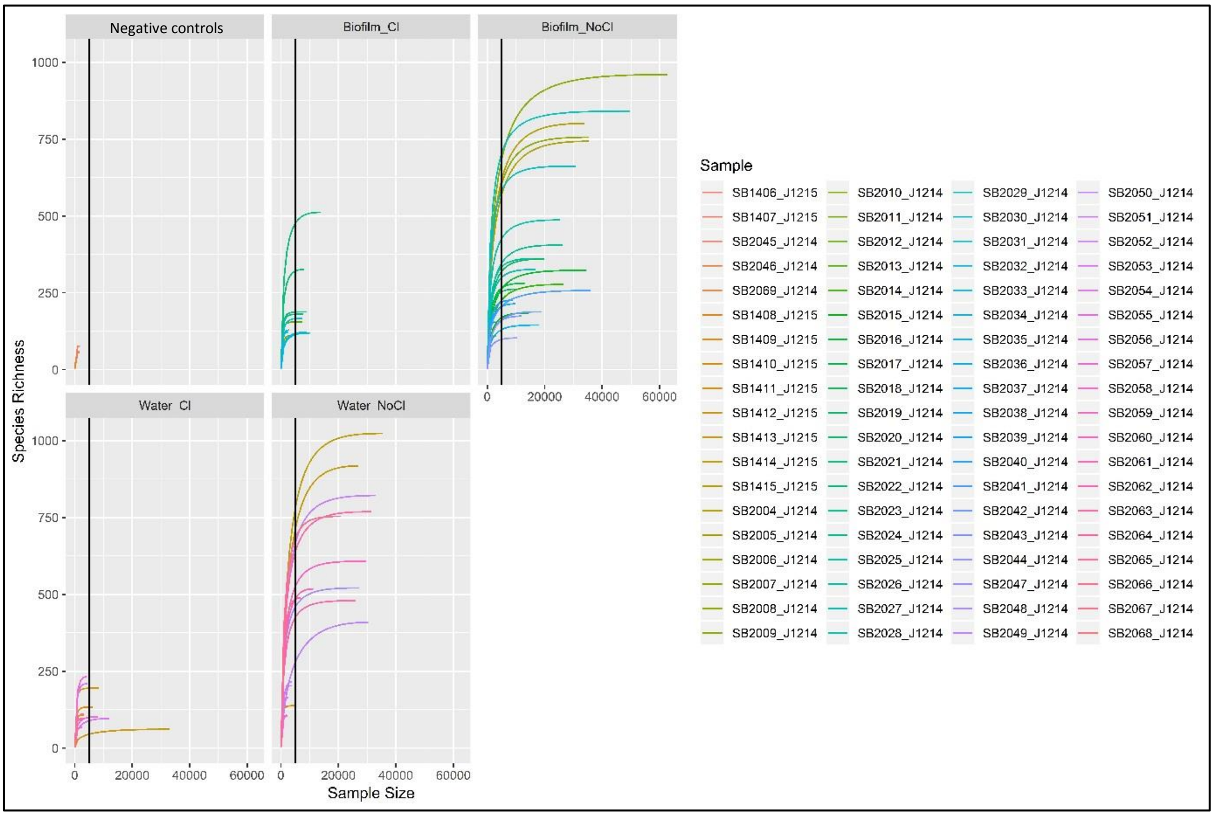

S1 Figure Legend: Rarefaction curves of 16s rRNA gene sequences of all samples grouped by negative controls (top left), chlorinated biofilm (top middle), non-chlorinated biofilm (top right), chlorinated water (bottom left) and non-chlorinated water (bottom right). The vertical line indicates the sequence number cut-off at 5,000 sequences. Samples with less than 5,000 sequences were excluded and the remaining samples rarefied to 5,259 sequences for subsequent ordinations and PERMANOVA analysis.
