# Supplementary material for: Opportunistic pathogens and large microbial diversity detected in source-to-distribution drinking water of three remote communities in Northern Australia
Source: PLoS Negl Trop Dis. 2019 Sep 5;13(9):e0007672. doi: 10.1371/journal.pntd.0007672 (PMC6728021; doi:10.1371/journal.pntd.0007672)
Supplement: S2 Fig — (PDF) [file pntd.0007672.s005.pdf]

S2 Figure:

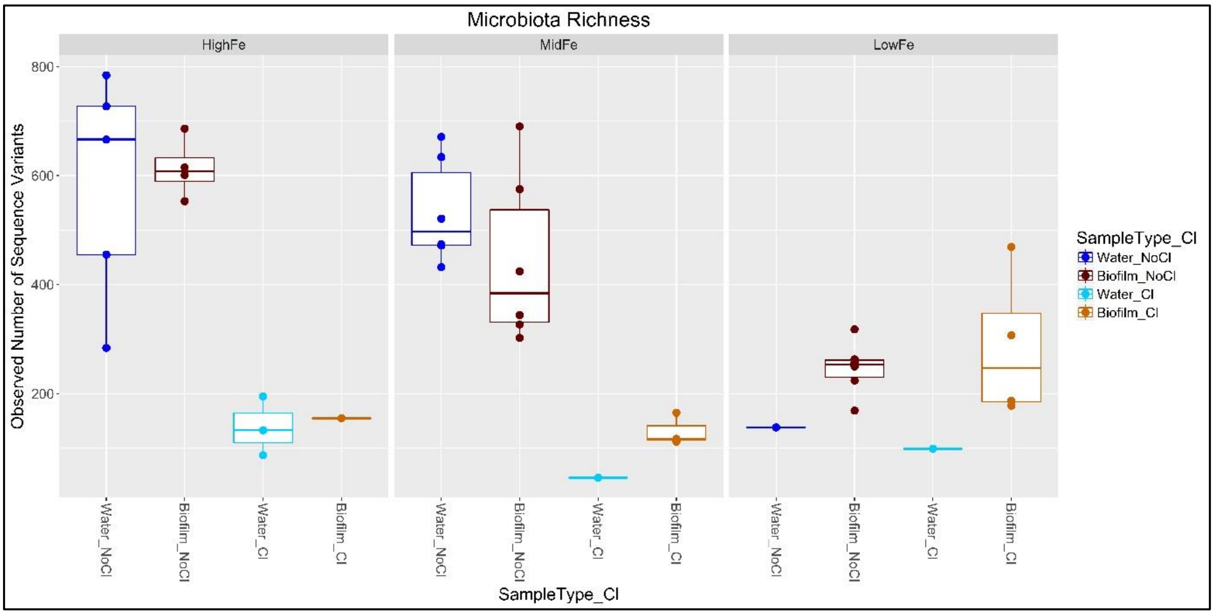

S2 Figure Legend: Microbial richness of water and biofilm samples in three remote communities and water supplies. Richness data are shown as observed number of sequence variants based on the final rarefied SV dataset.
