# Supplementary material for: Opportunistic pathogens and large microbial diversity detected in source-to-distribution drinking water of three remote communities in Northern Australia
Source: PLoS Negl Trop Dis. 2019 Sep 5;13(9):e0007672. doi: 10.1371/journal.pntd.0007672 (PMC6728021; doi:10.1371/journal.pntd.0007672)
Supplement: S3 Fig — (PDF) [file pntd.0007672.s006.pdf]

S3 Figure:

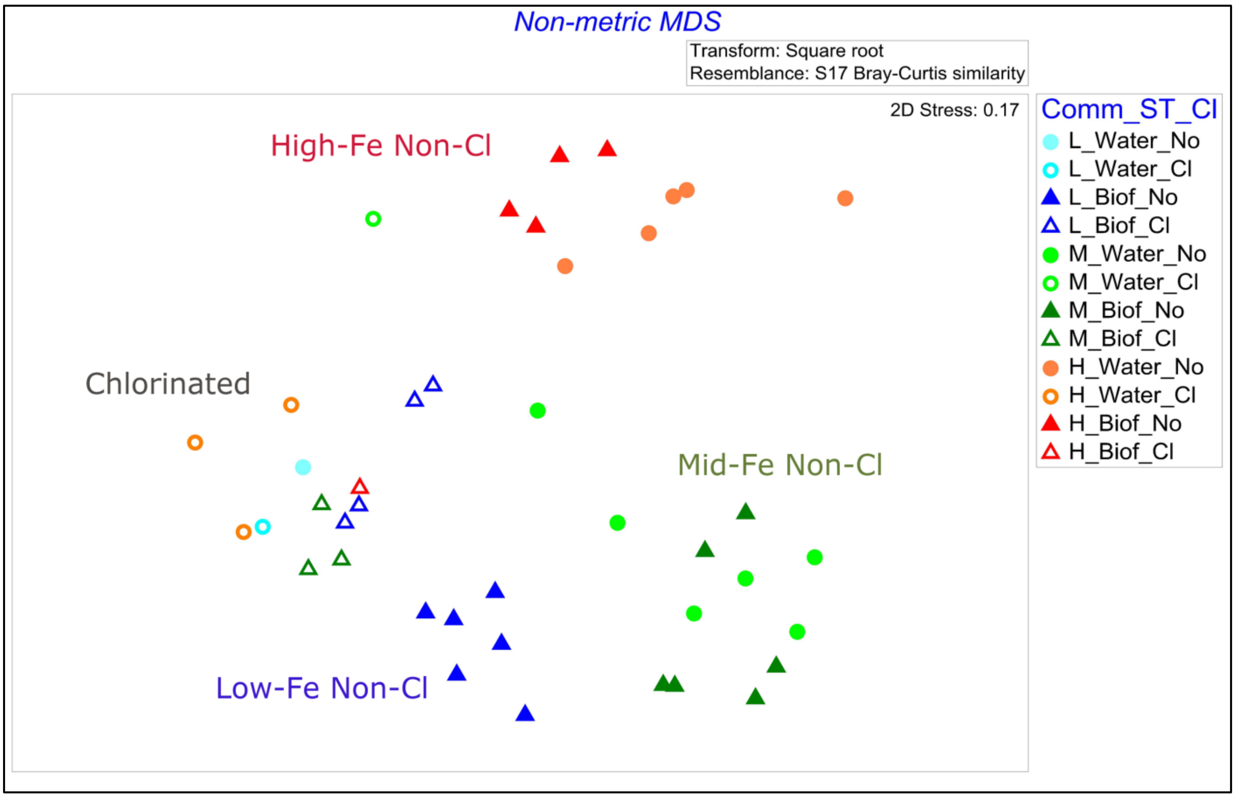

S3 Figure Legend: nMDS of microbiota in water supplies based on the Bray Curtis dissimilarity matrix of the square root transformed, rarefied SV abundance data. The nMDS stress value was 0.17.
