# Supplementary material for: Opportunistic pathogens and large microbial diversity detected in source-to-distribution drinking water of three remote communities in Northern Australia
Source: PLoS Negl Trop Dis. 2019 Sep 5;13(9):e0007672. doi: 10.1371/journal.pntd.0007672 (PMC6728021; doi:10.1371/journal.pntd.0007672)
Supplement: S4 Fig — (PDF) [file pntd.0007672.s007.pdf]

S4 Figure:

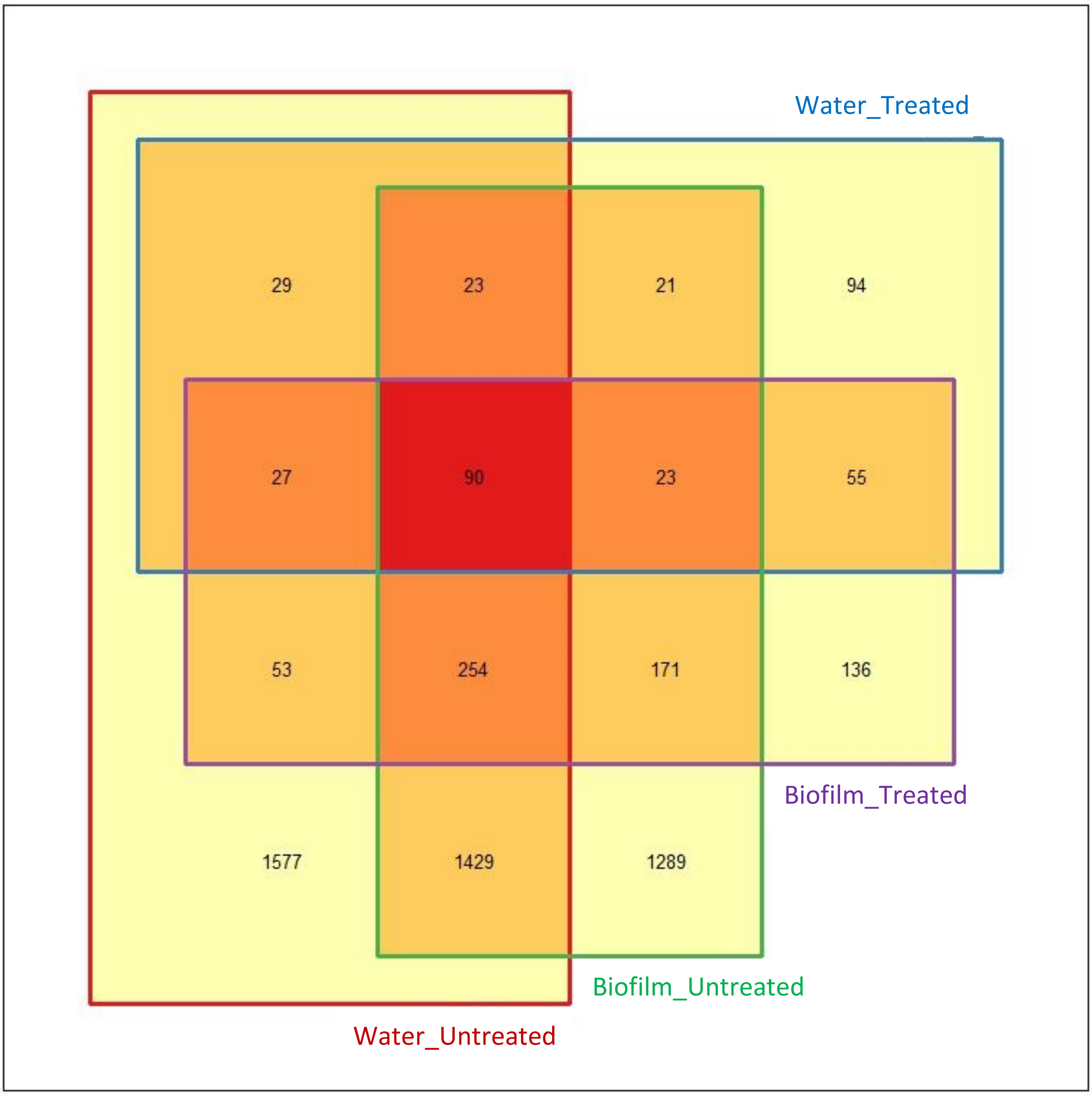

S4 Figure Legend: Venn diagram showing the number of SVs shared between water and biofilm, untreated and treated samples. The R package Vennerable was used to produce the Venn diagram. Only SVs which occurred in a minimum of two samples were considered.
