# Supplementary material for: Opportunistic pathogens and large microbial diversity detected in source-to-distribution drinking water of three remote communities in Northern Australia
Source: PLoS Negl Trop Dis. 2019 Sep 5;13(9):e0007672. doi: 10.1371/journal.pntd.0007672 (PMC6728021; doi:10.1371/journal.pntd.0007672)
Supplement: S5 Fig — (PDF) [file pntd.0007672.s008.pdf]

S5 Figure:

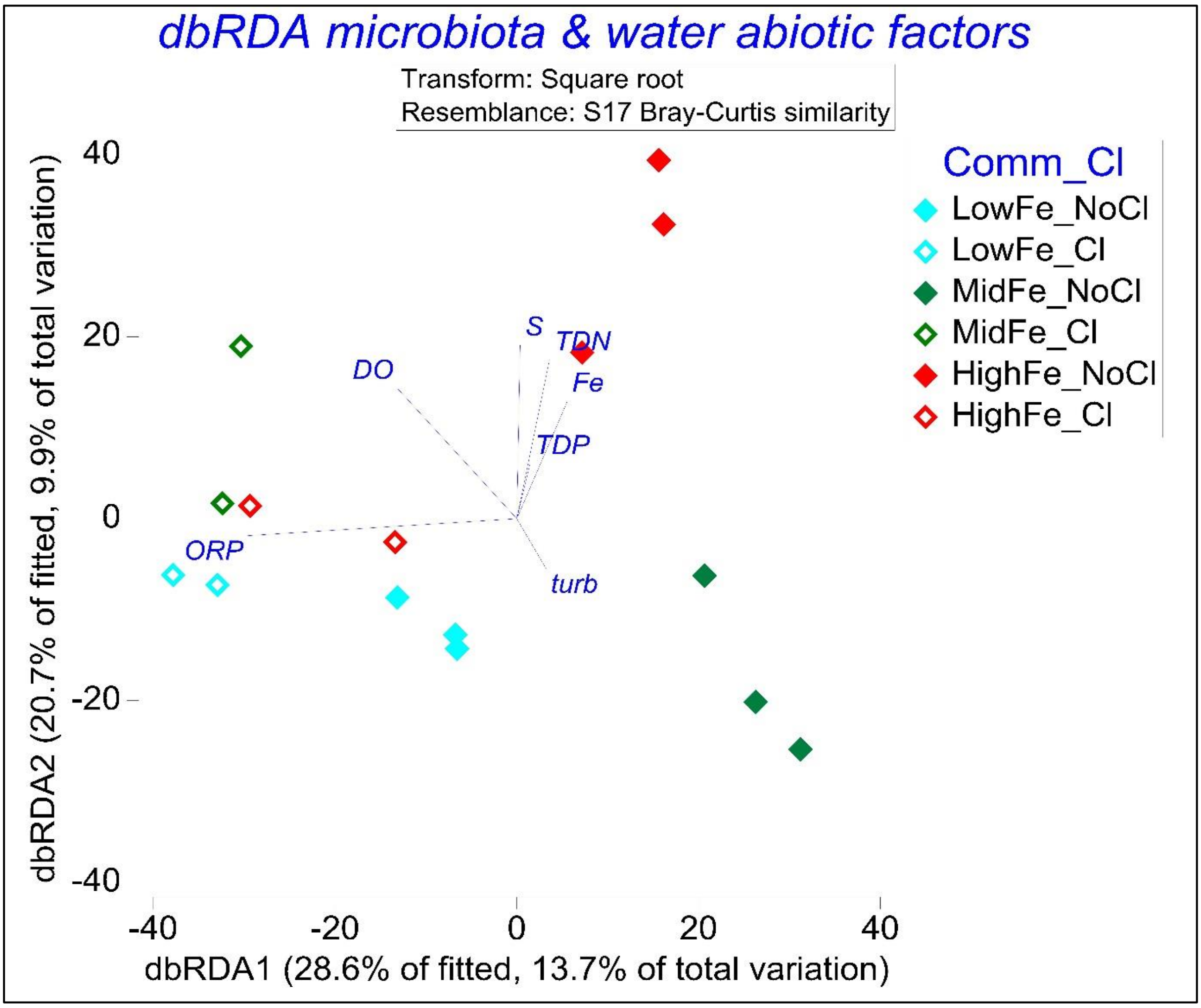

S5 Figure Legend: dbRDA showing the association between the microbiota in water and biofilms and water abiotic factors. The first two axes explained 23.6% of the total variation of the microbial composition. The dbRDA was based on a distance linear model with stepwise AICc selection. The length and direction of the vectors reflect the effect of the corresponding abiotic predictor on the dbRDA axes while accounting for all other predictors in the model. Chlorine levels, Mn, Mo, NOx and Ni were excluded due to collinearity.
