# Supplementary material for: Opportunistic pathogens and large microbial diversity detected in source-to-distribution drinking water of three remote communities in Northern Australia
Source: PLoS Negl Trop Dis. 2019 Sep 5;13(9):e0007672. doi: 10.1371/journal.pntd.0007672 (PMC6728021; doi:10.1371/journal.pntd.0007672)
Supplement: S6 Fig — (PDF) [file pntd.0007672.s009.pdf]

S6 Figure:

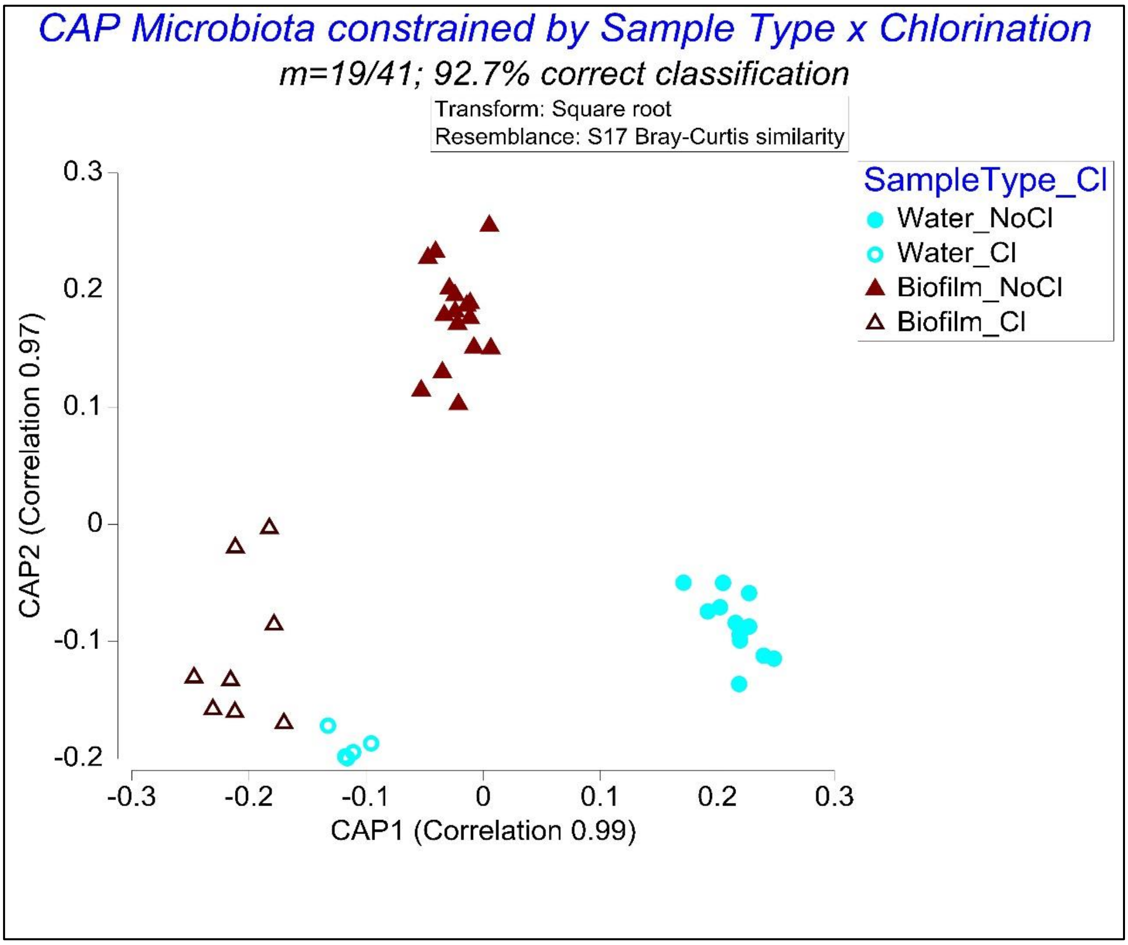

S6 Figure Legend: CAP analysis showing the microbiota constrained by sample type and chlorination status. Nineteen of 41 available PCO axes were used for the discriminant analysis resulting in the lowest error rate. Leave-one out cross validation showed an overall mis-classification error rate of 7.3%. All 16 non-chlorinated biofilm samples were classified correctly while one sample each of the other three categories was misclassified. The canonical correlations for each axis (0.99 and 0.97) reflect the strength of the association between the multivariate data cloud (microbiota samples) and the sample type x chlorination groups. A permutation-based test indicated significant differences in the positions of group centroids (P=0.001).
