# Supplementary material for: Opportunistic pathogens and large microbial diversity detected in source-to-distribution drinking water of three remote communities in Northern Australia
Source: PLoS Negl Trop Dis. 2019 Sep 5;13(9):e0007672. doi: 10.1371/journal.pntd.0007672 (PMC6728021; doi:10.1371/journal.pntd.0007672)
Supplement: S7 Fig — (PDF) [file pntd.0007672.s010.pdf]

S7 Figure:

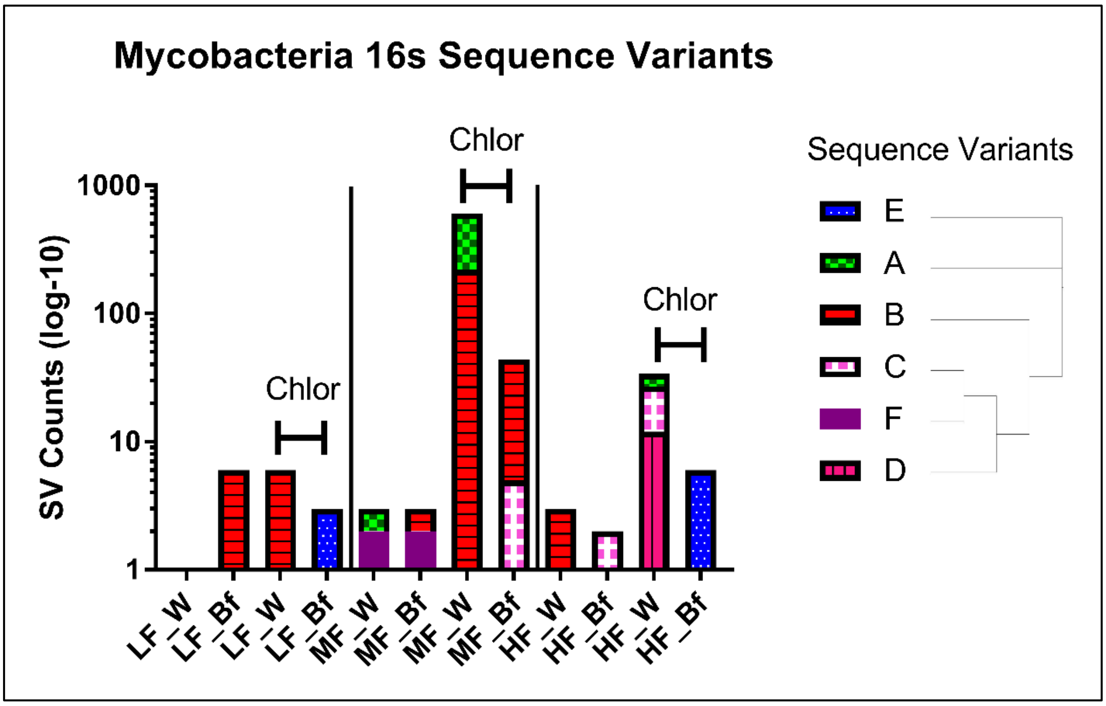

S7 Figure Legend: Mycobacteria SV counts (based on rarefied sequence dataset) across sample groups ("LF", "MF", "HF" LowFe, MidFe and HighFe water supply, "W" Water "Bf" Biofilm, "chlor" chlorinated or treated). The different shades and colours indicate different sequence variants. Their relatedness is indicated with a cladogram based on Neighbour-joining clustering. None of these SVs were detected in any negative controls.
